# Targeting cancer stem cells in medulloblastoma by inhibiting AMBRA1 dual function in autophagy and STAT3 signalling

**DOI:** 10.1007/s00401-021-02347-7

**Published:** 2021-07-24

**Authors:** Francesca Nazio, Agnese Po, Luana Abballe, Claudio Ballabio, Francesca Diomedi Camassei, Matteo Bordi, Antonio Camera, Simona Caruso, Ignazio Caruana, Marco Pezzullo, Caterina Ferraina, Giacomo Milletti, Matteo Gianesello, Sofia Reddel, Carmen Dolores De Luca, Donatella Ceglie, Sara Marinelli, Silvia Campello, Elena Papaleo, Evelina Miele, Antonella Cacchione, Andrea Carai, Maria Vinci, Enrico Velardi, Biagio De Angelis, Luca Tiberi, Concetta Quintarelli, Angela Mastronuzzi, Elisabetta Ferretti, Franco Locatelli, Francesco Cecconi

**Affiliations:** 1 Department of Paediatric Haematology and Oncology, Cell and Gene Therapy, Bambino Gesù Children’s Hospital, IRCCS, Rome, Italy; 2 Department of Molecular Medicine, Sapienza University, Rome, Italy; 3 Department of Experimental Medicine, Sapienza University, Rome, Italy; 4 Armenise-Harvard Laboratory of Brain Cancer, Department CIBIO, University of Trento, Trento, Italy; 5 Department of Laboratories, Pathology Unit, Bambino Gesù Children’s Hospital, IRCCS, Rome, Italy; 6 Department of Biology, University of Rome Tor Vergata, Rome, Italy; 7 Department of Maternal and Child Health, Sapienza University of Rome, Rome, Italy; 8 Consiglio Nazionale Delle Ricerche, Institute of Biochemistry and Cell Biology, Rome, Italy; 9 Computational Biology Laboratory, Danish Cancer Society Research Center, Copenhagen, Denmark; 10 Translational Disease Systems Biology, Faculty of Health and Medical Sciences, Novo Nordisk Foundation Center for Protein Research University of Copenhagen, Copenhagen, Denmark; 11 Department of Neuroscience and Neurorehabilitation, Neurosurgery Unit, Bambino Gesù Children’s Hospital, IRCCS, Rome, Italy; 12 Department of Translational Medical Sciences, University of Naples Federico II, Naples, Italy; 13 Department of Gynecology/Obstetrics and Paediatrics, Sapienza University, Rome, Italy; 14 Unit of Cell Stress and Survival, Danish Cancer Society Research Center, Copenhagen, Denmark

**Keywords:** Cancer stem cells, Autophagy, Brain tumours, Therapy

## Abstract

Medulloblastoma (MB) is a childhood malignant brain tumour comprising four main subgroups characterized by different genetic alterations and rate of mortality. Among MB subgroups, patients with enhanced levels of the c-MYC oncogene (MB_Group3_) have the poorest prognosis. Here we identify a previously unrecognized role of the pro-autophagy factor AMBRA1 in regulating MB. We demonstrate that AMBRA1 expression depends on c-MYC levels and correlates with Group 3 patient poor prognosis; also, knockdown of AMBRA1 reduces MB stem potential, growth and migration of MB_Group3_ stem cells. At a molecular level, AMBRA1 mediates these effects by suppressing SOCS3, an inhibitor of STAT3 activation. Importantly, pharmacological inhibition of autophagy profoundly affects both stem and invasion potential of MB_Group3_ stem cells*,* and a combined anti-autophagy and anti-STAT3 approach impacts the MB_Group3_ outcome. Taken together, our data support the c-MYC/AMBRA1/STAT3 axis as a strong oncogenic signalling pathway with significance for both patient stratification strategies and targeted treatments of MB_Group3_.

**Supplementary Information:**

The online version contains supplementary material available at 10.1007/s00401-021-02347-7.

## Introduction

Medulloblastoma (MB) is the most common malignant paediatric brain tumour localized in the hindbrain. Based on transcriptional profiling studies, it is now clear that MB is a heterogeneous disease and multiple distinct molecular subgroups have been identified [65, 66, 93]. Two of them are associated with alteration in the Wingless (WNT) and Sonic Hedgehog (SHH) developmental signalling pathways (the WNT and SHH subgroups), while the other two are less well molecularly characterized and referred to as Group 3 and Group 4 tumours. Using genome-wide DNA methylation and gene expression analyses, 12 different subtypes of MB have been recognized, highlighting the heterogeneous and complex nature of disease subgroups [13, 64]. More recently, using multiple complementary bioinformatic approaches, a study refined Group 3/4 classification, identifying eight subtypes (I–VIII), and describing a more complex intratumoral heterogeneity within Group 3 and 4 [39, 84]. Although multimodal treatments currently in use resulted into improved survival of MB patients, this was obtained at the price of severe side effects, including cognitive deficits, endocrine disorders, and, later in life, increased incidence of secondary cancers [30, 70, 71, 76]. Recent studies identified features predictive of dismal outcome in MB patients: they include amplification or overexpression of the MYC oncogene, < 3 years of age, subtotal resection (STR defined as leaving > 1.5 cm^2^ of residual tumour), dissemination at diagnosis and large-cell/anaplastic histology (LCA) [67, 79]. A large body of evidence reveals that key properties related to MB aggressiveness are: stemness, invasion capacity, and cell motility [40]. All these features have been associated with the presence of cancer stem cells (CSCs) also in MB [87, 88]. MB stem cells (MBSCs) can be identified as CD133/Prom1-positive cells that show a marked capacity for proliferation, self-renewal, and differentiation in vitro [87]. Autophagy, a process of resolving and recycling proteins and damaged cellular organelles, protects cells from nutritional deficiency and can prevent cell death [53]. Importantly, multiple studies have clearly demonstrated a link between autophagy and cancer [54, 89]. More recently, it has been suggested that both autophagy inhibition and autophagy activation represent potential approaches to increase CSC sensitivity to therapy [62]. Indeed, regulatory factors acting at different steps of the autophagy process have been identified as able to deliver both oncogenic and tumour-suppressive signalling pathways of cancer [53, 97]. Autophagy and beclin 1 regulator 1 (AMBRA1) is a pro-autophagy protein involved in many and diverse functions, spanning from autophagy upstream regulation and cell proliferation control to neurogenesis and embryonic development [4, 18, 19, 29, 58, 63, 92]. Up to now, several roles for AMBRA1 and autophagy have been described in human cancer; however, its function in the origin of brain tumours, such as MB, and in lineage-specific mechanisms that could regulate stem cell behaviour is largely unknown.

Here, we demonstrate a role for AMBRA1 and autophagy in MB growth and dissemination, with a focus on MBSCs. We show that AMBRA1 is differentially expressed in MB subtypes, depending on c-MYC and Myc-Interacting Zinc Finger Protein 1 (MIZ-1) expression. Inhibition of AMBRA1 affects both autophagy and STAT3 signalling and induces MB differentiation, reduction of migration and loss of stem cell potential. Indeed, STAT3 inhibition has already been proposed as a therapeutic strategy for brain tumours [51], despite its role in autophagy induction [104]; here, we propose that combined inhibition of both autophagy and STAT3 impairs MB growth.

In sum, analysing different MB cell lines, primary tumour samples, orthotopic xenograft models, and an immunocompetent MB_Group3_ mouse model, we demonstrate a novel mechanism of action for AMBRA1 in the control of MB_Group3_ oncogenesis through regulation of both STAT3 signalling and autophagy.

## Methods

### Cell culture

DAOY, D283-Med and CHLA-01-Med cells were cultured according to the recommended conditions (ATCC). Cells were maintained in humidified atmosphere containing 5% CO_2_ at 37 °C and mycoplasma test was performed every 3 months.

Human MB stem-like cells (hCSC) were derived from primary human MB SHH as previously described [1]. Human stem-like cells were cultured in Selective medium (SM) for stem cells enrichment, containing DMEM/F12 (Gibco) supplemented with 0.6% glucose, 25 μg/ml insulin, 60 mg/ml *N*-acetyl-l-cysteine, 2 mg/ml heparin, 20 ng/ml EGF, 20 ng/ml basic FGF (Peprotech, Rocky Hill, NJ), penicillin–streptomycin, and B27 supplement without vitamin A (Gibco). All the cell lines were regularly monitored for mycoplasma contamination.

Cerebellar granule neuron precursors (GNPs) (prepared from CD1 4‐day‐old mice) were isolated and cultured as previously described [6, 22]. When indicated, GNPs were treated with Shh (3 μg/ml) for up to 72 h (R&D, Minneapolis, MN, USA).

Cerebellar Neural Stem Cells (cNSCs) were obtained from cerebella of postnatal 4-day-old wild-type black 6/C56 (C57BL/6) mice (Charles River) and were derived as previously described [11].

Neural Stem Cells (NCSs) were extracted from Medial Ganglionic Eminences at E14.5 as described elsewhere [58]. NSCs were dissociated mechanically into a single-cell suspension and cultured in Selective medium (SM) for stem cells enrichment, containing DMEM/F12 (Gibco) supplemented with 0.6% glucose, 25 µg/ml insulin, 60 mg/ml *N*-acetyl-l-cysteine, 2 mg/ml heparin, 20 ng/ml EGF, 20 ng/ml FGF basic (Peprotech, Rocky Hill, NJ), Zell-Shield (Minerva Biolabs), and B27 supplement (Gibco).

### RNA sequencing

Next generation sequencing experiments on D283-Med cells, including samples quality control, were performed by Genomix4life (Baronissi, Salerno, Italy). RNA concentration in each sample was assayed with a ND-1000 spectrophotometer (NanoDrop) and its quality assessed with the Agilent Tapestation 4200 (Agilent Technologies). Indexed libraries were prepared from 1 μg/ea purified RNA with TruSeq Stranded mRNA Sample Prep Kit (Illumina) according to the manufacturer’s instructions. Libraries were quantified using the Agilent TapeStation 4200 (Agilent Technologies) and pooled such that each index-tagged sample was present in equimolar amounts, with final concentration of the pooled samples of 2 nM. The pooled samples were subjected to cluster generation and sequencing using an Illumina Nextseq 500 (Illumina) in a 2× 75 paired-end format at a final concentration of 1.8 pmol. The raw sequence files generated (.fastq files) underwent quality control analysis using FastQC Fastq underwent Quality Control using FastQC tool (http://www.bioinformatics.babraham.ac.uk/projects/fastqc/). The mapping of paired-end reads was performed using STAR (version 2.5.2a) [25] on reference genome assembly hg19 obtained from Ensembl (GRCh37.p13) [41] (https://grch37.ensembl.org/Homo_sapiens/Info/Index). The quantification of transcripts expressed for each sample was performed using FeatureCount [55], algorithm. DESeq2 [77] was used to normalize the data and then to perform the differential expression analysis.

For CHLA-01-Med cells, RNA integrity was analyzed with Agilent 4150 Bioanalyzer (Agilent Technologies, Santa Clara, CA, USA). RNA concentration was tested by Qubit RNA Assay Kit in Qubit Fluorometer (Invitrogen, Carlsbad, CA, USA). RNA integrity numbers (RINs) were ≥ 8.7.RNA-seq libraries were prepared using the Illumina^®^ RNA Prep, Tagmentation (L) with Enrichment kit (Exome Panel) (Illumina, San Diego, CA, USA) that generated enriched libraries for dual-indexed, paired-end sequencing, according to the manufacturer’s instructions. Briefly, a total amount of 100 ng RNA per sample was used for reverse transcription and the resulted cDNA was tagmented with enrichment bead-linked transposomes and then amplified to add indexes and adapters. Two-hundred ng of the resulting libraries were normalized for three-plex enrichment step consisting of a hybridization with capture probes that delivers comprehensive coverage of coding RNA sequences target regions (no. of target genes 21,415, no. of target exonic regions 214,126 no. of probes 425,437 RefSeq exome percent covered 98.3%). Enriched libraries were then amplificated and checked at Agilent 4150 Bioanalyzer for the correct average size (~ 420 bp).

Finally, 1 pM of the final pooled libraries were subjected to paired-end sequencing with pair end 101 bp reading length on an Illumina NextSeq 550 sequencer (Illumina, San Diego, CA, USA). 1% of Phix 1 pM was used as internal sequencing control. Fastq files were uploded on Base Space Sequence Hub (Illumina, San Diego, CA, USA) and DRAGEN RNA pipeline and DRAGEN differential expression apps were launched for reads quality check, hg38 alignment and differential expression analysis.

### Data analysis

The gene-set enrichment tool Enrichr [15, 49, 102] was used to perform functional annotation analysis of KEGG pathways.

Gene Set Association Analysis (GSAA) of the expression data was used to assess enrichment of selected gene lists, indicated respectively in the figure legend, derived from KEGG PATHWAY Database. The list of transcriptional factors described to be essential for stemness was derived from this review [35]. We used the GSAASeqSP tool, a Java-based desktop application (software GSAA 2.0), according to the manufacturer’s instructions [103]. We have used the default setting choosing Signal2Noise_log2Ratio for differential expression analysis of individual genes.

Venn diagram is made using BioVenn tool (BioVenn—a web application for the comparison and visualization of biological lists using area-proportional Venn diagrams) [42].

The data discussed in this publication are accessible through GEO Series accession number GSE134109.

### MB specimens and molecular subgrouping

Surgical specimens of primary MB were collected at the Bambino Gesù Children’s Hospital in Rome, with Institutional Review Board approval. MB specimens were obtained from a cohort of 47 patients with histologically confirmed diagnosis who had undergone surgical resection at the Bambino Gesù Children’s Hospital in Rome between 2010 and 2017. All specimens were formalin-fixed, sectioned, stained with haematoxylin and eosin (H&E) and examined through microscopy according to the international staging system for paediatric brain tumours [56]. Clinical features and sample treatments are detailed in Supp. Table S1. Molecular characterization of MBs was performed as previously described [65].

RNA from normal cerebellum was purchased from Biochain Institute (*n* = 4: R1234039-50, Total RNA-Human Brain cerebellum Adult; *n* = 4: R1244041-50 and R1244040-50, Total RNA-Human fetal Brain cerebellum).

### Histologic and IHC data

**Fig. 1:**
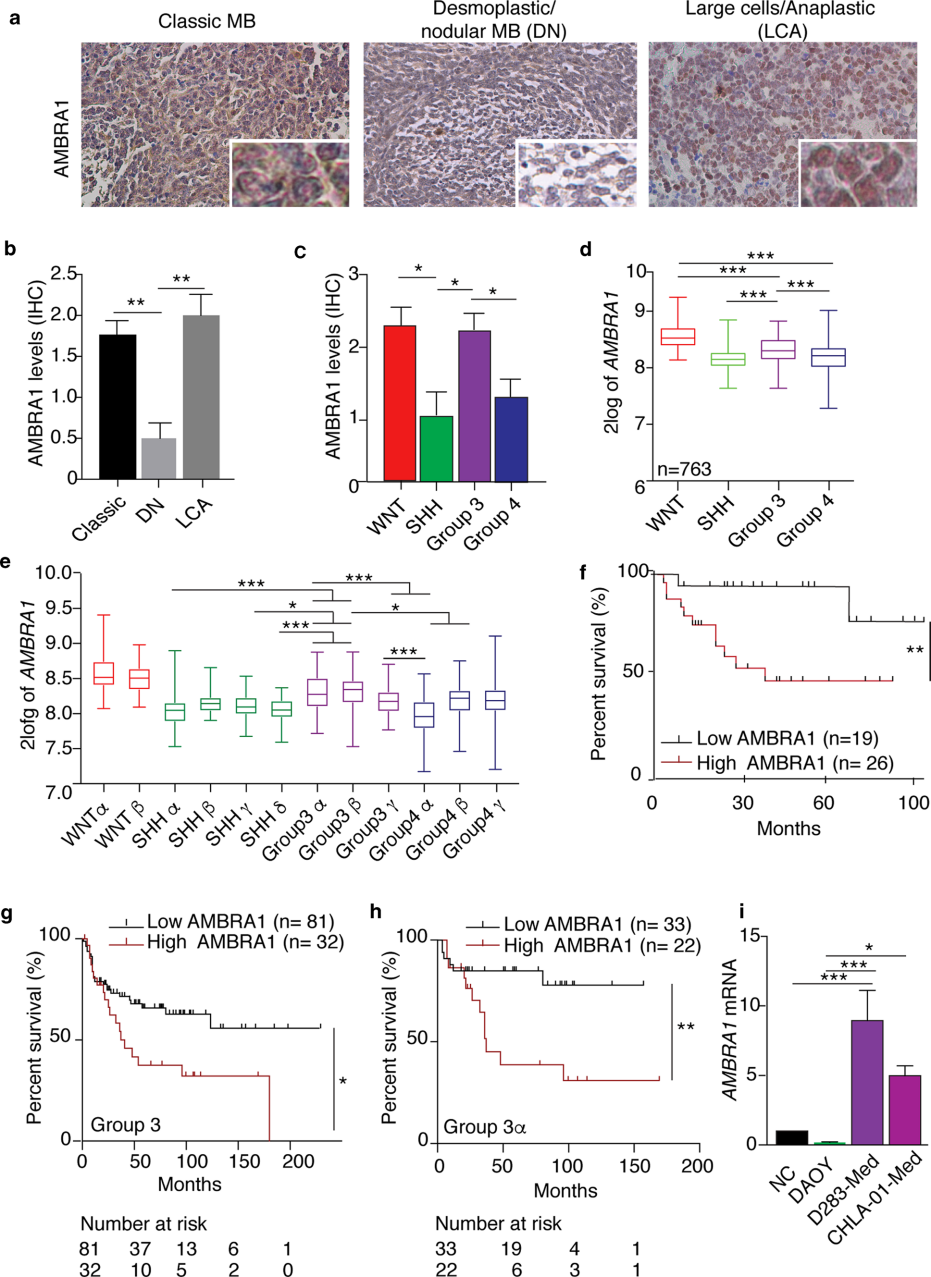
AMBRA1 expression correlates with patient survival in MB. **a–c** Representative IHC images in high magnification fields (40 ×) showed AMBRA1 expression in human MB subtypes. The staining levels could be ranked in three grades, shown as negative (score 0), weak positive (score 1), median positive (score 2), and strong positive (score 3). Right, statistical graphs showed that staining levels of AMBRA1 among histological (classic *n* = 30, desmoplastic/nodular (DN) *n* = 7 and large cells/anaplastic (LCA) *n* = 10 MBs; **p < 0.01) and molecular MB subgroups (WNT *n* = 8, SHH *n* = 9, Group 3 *n* = 13, Group 4 *n* = 16; *p < 0.05). Data were analysed by one-way analysis of variance (ANOVA) followed by Tukey post hoc test. **d** RNA log2 expression of *AMBRA1* derived from both the publicly available dataset Cavalli (763 samples, fpkm normalized, mb500rs1 chip), grouped according to the molecular subgroup disease variants. Data were analysed by one-way analysis of variance (ANOVA) followed by Tukey post hoc test (***p < 0.001). **e** RNA log2 expression of *AMBRA1* derived from the publicly available dataset Cavalli (763 samples, fpkm normalized, mb500rs1 chip), grouped according to the 12 molecular subtype disease variants. Data were analysed by one-way analysis of variance (ANOVA) followed by Tukey post hoc test (*p < 0.05, ***p < 0.001). **f** Kaplan–Meier analysis for our cohort of MB patients according to AMBRA1 expression levels. Low AMBRA1 *n* = 19; High AMBRA1 *n* = 27; *p* = 0.01. *p* value was determined by the log-rank (Mantel- Cox) test. **g** Kaplan–Meier analysis for MB_Group3_ patients according to AMBRA1 expression levels in Cavalli dataset. Low AMBRA1 *n* = 81; High AMBRA1 *n* = 32. *p* = 0.026; expression cut-off 338.000. **h** Kaplan–Meier analysis for G3α patients according to AMBRA1 expression levels in Cavalli dataset. Low AMBRA1 *n* = 33; High AMBRA1 *n* = 22. *p* = 0.010; expression cut-off 321.400. **i** qPCR analysis of *AMBRA1* mRNA in normal cerebellum (NC) and different MB cell lines. Both *GADPH* and *B2M* were used as internal control. Data are expressed as the mean value ± SEM (*n* = 6). NC was arbitrarily defined as 1.00. Data were analysed by one-way analysis of variance (ANOVA) followed by Tukey post hoc test (*p < 0.05; ***p < 0.001)

All samples of human tissue research in this study were collected at the Bambino Gesù Children’s Hospital in Rome, with Institutional Review Board approval. These clinical MB specimens were examined and diagnosed by pathologists. Tissue sections of clinical specimens were stained with antibodies against AMBRA1 (1:100, 26190002 Novus) and CD133 (1:100, #86781 Cell Signalling). The data shown in Fig. 1b, c were generated based on the scores of IHC antibody quantified as follow: 3 + , positive signals in > 50% tumour cells; 2 + , positive signals in > 25% tumour cells; 1 + , positive signals in > 5% to 25% tumour cells; 0%, low or no positive signals in < 1% tumour cells [3, 105]. Tumors with 0 and 1 staining were considered as low expression and tumors with 2 to 3 scores were considered as high expression. The analyses were assessed by an experienced pathologist (F.D.C.), blinded to sample identity and were expressed as the mean percentage of immunostained tumour cells. Kaplan–Meier survival analyses were used to assess the relevance and importance of AMBRA1 in human MB tumors. In the analyses of two datasets, the upper-quartile or upper-third samples were defined as AMBRA1-high, and the rest of MB samples were considered as AMBRA1-low**.**

### Analyses of public available transcriptomics data

We used the R2 genomics platform (http://r2.amc.nl) to identify suitable transcriptomics datasets with molecular and histological subtypes available for MB. We selected the following datasets: Pfister (167 samples, fpkm normalized, mb500rs1 chip) and Cavalli (763 samples, rma_sketch normalized, hugene11t chip). For each of them, we download log_2_ transformed and normalized data through the *data grabber* function for each subgroup (SHH, Group4, Group3 and WNT) and subtype (I–VIII or α, β, γ, δ) for AMBRA1 (52731_at probe) and MYC (202431_s_at probe) expression levels.

We performed a Q–Q plot analysis and the Shapiro–Wilk’s method to perform a normality test of the data distribution. When a minority of the distributions was not approximable to a normal distribution, we employed a non-parametric ANOVA test (Kruskal–Wallis test) to assess the significance of the differences in expression levels of the same gene in the different subtypes.

The correlation analyses between AMBRA1, c-MYC and CD133 in both Cavalli and Pfister datasets were also carried out through R2 (Genomics Analysis and Visualisation Platform; http://r2.amc.nl).

Survival analysis was generated using the dataset GSE85217 (Cavalli). AMBRA1 and c-MYC gene expression levels were expressed as a binary variable using the cut-off value generated by the Kaplan Scan online algorithm (https://hgserver1.amc.nl/cgi-bin/r2/main.cgi?option=kaplanscan1). The overall survival right-censored at 5 years, was estimated using the Kaplan–Meier method and *p* values were assessed using the log-rank test to express statistical differences in overall survival among patients in MB_Group3_ and in G3α. Cox proportional hazards regression analysis was performed to determine the independent effects of AMBRA1 and c-MYC gene expression levels on overall survival in MB_Group3_ and in G3α. Statistical analyses were performed using R v. 4.0.3 (www.r-project.org) and graphics were generated using GraphPad Prism 8 (www.graphpad.com).

### Expression constructs, siRNAs, and chemical reagents

Plasmids coding for c-MYc-MYC^V394D^, MIZ-1^WT^ and MIZ-1^ΔPOZ^ were purchased from Addgene (#74162, #74167, #74160). FLAG-STAT3 plasmid is a gift of M. Jäättelä. AMBRA1 plasmids were constructed as previously described [29]. DAOY cells were transfected using Lipofectamine 2000 in accordance with the manufacturer’s instructions (ThermoFisher Scientific). Both D283-Med and CHLA-01-Med were transfected using Lipofectamine 3000 in accordance with the manufacturer’s instructions (ThermoFisher Scientific). RNA interference was performed using Lipofectamine RNAiMAX reagent in accordance with the manufacturer’s instructions (ThermoFisher Scientific).

siRNA oligoribonucleotides corresponding to *AMBRA1* cDNA sequence were purchased from Dharmacon as previously reported [19, 29]; siRNA against MIZ-1 was purchased from Santa Cruz Biotec. (sc-38085); Stealth siRNAs (novel modified siRNA with enhanced stability) were purchased from ThermoFisher Scientific against c-MYC (#1 HSS106839; #2 HSS181389); STAT3 (VHS40491); SOCS3 (#1HSS113311; #2HSS113312; #3HSS113313). BECLIN 1 and ATG7 were described previously [19, 37]; for the measurement of autophagy flux, Chloroquine (CQ) (Sigma-Aldrich) was used at 20 μM for 1 h.

NSC 74859 was purchased from Tocris Bioscience. WP1066 was purchased from MCE (MedChem Express).

### Lentiviral production and infection

Lentiviral vectors were produced by transfecting human embryonic kidney (HEK) 293T cells with the AMBRA1 shRNA construct using Lipofectamine 2000 in accordance with the manufacturer’s instructions (ThermoFisher Scientific). After removal of cell debris with filter, the supernatant containing lentivirus was used to infect MBSCs. Short hairpin (shRNA) mediated knock down of endogenous AMBRA1 was performed using PLKO lentivirus carrying AMBRA1 shRNA (Sigma-Aldrich, TRNC0000168652) or non-targeting shRNA as control (Sigma-Aldrich, SHC003V). After 72 h cells were harvested and subjected to downstream analyses.

### Cell proliferation and colony formation assay

Cell proliferation was assessed by MTS (3-(4,5-dimethylthiazol-2-yl)-5-(3-carboxymethoxyphenyl)-2-(4-sulfophenyl)-2H-tetrazolium) assay after 72 h of seeding. D283-Med cells were knocked-down for AMBRA1 or with control as specified in the RNAi section or treated with CQ as indicated. They were seeded in 96-well plates (5000 cells in each well) and during the last hour MTS was added to the medium. Each combination of cells was seeded in triplicate wells and analysed at least three times. The MTS assay was analysed spectrophotometrically at 490 nm using a 96-well plate reader.

Cell numbers for D283-Med cells after AMBRA1 downregulation was determined by trypan blue exclusion at 72- and 96-h post transfection, respectively.

For the medullosphere (MS) forming assay, human MBSCs and CHLA-01-Med cells were disaggregated to single cell through dissociation solution non-enzymatic buffer (Cod: C5789, Sigma-Aldrich). hMBSCs were plated at clonal density (1–2 cells/mm^2^) into 96-well plates, while CHLA-01-Med were plated at the density of 70 cells/well into 96-well plates. After 10–14 days, MS were counted and divided by the number of cells plated to determine the percentage of MS forming cells, respectively.

For soft agar assay, D283-Med cells were knocked-down for AMBRA1 or with control as specified in the RNAi section and after 24-h tumour cells were resuspended in MEM medium with 0.5% agar and plated into 6-well plates at 3000 cells/well on top of a precast semisolid 1% agar underlayer. Colonies of > 50 cells were scored after 1 week of growth.

To produce MS, DAOY cells were grown at confluence in adhesive condition, trypsinized, pelleted and plated (6 × 10^4^/ml) in ultra-low attachment T25 Flasks (Corning) for 3 and 7 days in serum-free EUROMED CSC Neuronal medium. After the first passage (P1) MBs were spun down at 1400 rpm (368 g), harvested and resuspended as a single cells suspension in the same serum-free medium at the same concentration to obtain subsequent passages. The MS were identified as spherical ovoid aggregates with smooth outlines of more than 20 cells under microscope observation.

### Migration assays

A migration assay was carried out using Boyden chambers with 8-μm pore polycarbonate membrane transwell chambers (Millipore, Merck). D283-Med cells were transfected with AMBRA, ATG7, STAT3 siRNAs or negative control siRNA respectively or with CQ 40 μM for 48 h. Forty-eight hours after transfection, D283-Med cells were washed and re-suspended in media without FBS for 18 h. Cells were then trypsinized and 150,000 cells plated into the top chamber of the transwell in a volume of 300 μl; while 500 μl of complete media was added to the bottom chamber. Cells were allowed to migrate for 16–18 h at 37 °C. Then, migrated cells present in the bottom of the transwell were stained with WST-1 reagent (1:10, Millipore) and quantified by colorimetric measurement at 420–480 nm using a spectrophotometer.

### Invasion assays

To evaluate the invasion capacity of D283-Med cells in vitro*,* we used the QCM cell invasion assay (Millipore, Merck) following the manufacturer’s instructions. 10% FBS was used as a chemo attractant in the lower chamber. All experiments were performed in quadruplicate. Data are expressed as optical density (570 nm) of invasive cells. Briefly, D283-Med cells were transfected with AMBRA, ATG7, STAT3 siRNAs or negative control siRNA respectively or with CQ 40 μM for 48 h. After 48 h, cells were plated at 2.5 × 10^5^ cells/well in the upper chamber without FBS. Chambers were removed 24 h later and stained following the manufacturer’s instructions.

### Chromatin immunoprecipitation

ChIP was performed using the MAGnify(TM) Chromatin Immunoprecipitation System (ThermoFisher Scientific) as previously described [11]. Briefly, for each ChIP reactions 250,000 cells were used; cross-linking was performed using 1% formaldehyde for 10 min at room temperature, followed by treatment with 0.125 M glycine for 10 min at room temperature to stop the reaction. Cells were then lysed with the lysis buffer provided and chromatin was fragmented using the Bioruptor sonicator (Diagenode) for three cycles of 10 min of 30 s on and 90 s off to obtain fragments of about 400–600 nucleotides. The primary antibody was coupled for 1 h to the protein A/G Dynabeads, then the chromatin was added to their respective antibody/beads for 2 h. After washing passages and de cross-linking, DNA was purified and amplified with primers listed below.

The following antibodies were used at the concentration of 5 μg: IgG rabbit (ThermoFisher Scientific), IgG mouse (ThermoFisher Scientific), mouse monoclonal anti-Miz-1 (B-10, sc-136985), rabbit polyclonal anti-c-Myc (Cell signalling, code: 9402) and rabbit polyclonal anti-acetyl-histone 3 (Millipore, code: 06599). Eluted DNA was qPCR amplified with primers designed in the human AMBRA1 promoter and the ChIP-qPCR data were analysed relative to percentage of input. Primers were designed with Primer-Blast designing tool (http://www.ncbi.nlm.nih.gov/tools/primer-blast/) and Primers tool (Genomatix Genome Analyzer, GGA, v3.30126, https://www.genomatix.de/) and are reported below:

AMBRA1 Fw: 5′-CCGTGGCTTATGATCTGGGT-3′

AMBRA1 Rev: 5′-ATCGTTCAAGGAACCACAGC-3′

### Luciferase assay

PCR fragment of the human *Ambra1* promoter (1 kb) spanning the MIZ-1-binding site [99], covering the start sites of transcription, is cloned in a pEZX-LvPG04 plasmid (GeneCopoepia) in front of a luciferase open-reading frame and performed transient reporter assays. NEG-LvPG04 and GAPDH-PG04 plamids (GeneCopoepia) are used as negative and positive controls, respectively. HEK293 cells were transiently transfected with the reporter constructs together with MIZ-1^WT^, MIZ-1^ΔPOZ^, c-MYC^WT^ and c-MYC^V^^394D^ expression plasmids respectively or empty vector as control. Cells were harvested after 48 h, gaussia luciferase (GLuc) and secreted Alkaline Phosphatase (SEAP) luciferase activity are measured according to the manufacture's protocol (LF031, GeneCopoepia). SEAP is available on the same vector of GLuc and is used for transfection normalization.

### RNA isolation and quantitative real-time PCR (qPCR)

Cells were washed twice with cold PBS and lysed for total RNA extraction using a Qiagen RNeasy Kit. Extracted RNAs were then used to generate cDNA using MLV reverse transcriptase (Promega). Real-time PCR was carried out in triplicate using SYBR Green PCR Master Mix (ThermoFisher Scientific) on an Applied Biosystem Real-Time. The primers were used in this study are reported in Supp. Table S2.

### Immunoblot analysis (WB)

Cells were lysed in a RIPA buffer (50 mM Tris–HCl, pH 8.0/150 mM Sodium chloride/1% NP-40/0.5% sodium deoxycholate/0.1% sodium dodecyl sulfate/2 mM EDTA) containing 1 × protease and 1 × phosphatase inhibitor cocktails (Sigma-Aldrich). Protein samples were quantified using the Bradford assay reagent (Bio-Rad) in accordance with the manufacturer instructions. Protein samples were subjected to SDS-PAGE and transferred to polyvinylidene fluoride (PVDF) membranes in 25 mM Tris, 192 mM glycine. Membranes were incubated with indicated antibodies for overnight at 4 °C. Following wash with PBS-T (PBS containing 0.1% Tween-20), the blot was incubated with corresponding peroxidase-labelled secondary antibodies (1:200). Blots were developed with enhanced chemiluminescence (ECL, Millipore) reaction according to manufacturer instructions. The primary antibodies used in this study were: AMBRA1 (sc-398204 mouse monoclonal antibody Santa Cruz Biotechnology), Vinculin (sc-73614 mouse monoclonal antibody Santa Cruz Biotechnology), Ambra1 (ABC131 Merck Millipore), c-MYC (#5605 rabbit polyclonal antibody Cell Signaling, c-Myc (9E10): sc-40 Santa Cruz Biotechnology), MIZ-1 (sc-136985 Santa Cruz Biotechnology), CD133 (#86781 Cell Signaling), NESTIN (ab 22035 Abcam), p62 (sc-28359 polyclonal antibody Santa Cruz Biotechnology), LC3B (D11 #3868 Cell Signaling), p-ATG14 (#13155 Cell Signaling), SYNAPTOPHYSIN (ab 32127-rabbit monoclonal antibody Abcam), cleaved-PARP (#9541 Cell Signaling), GFAP (ab7260 Abcam), FOXO3a (#2497 Cell Signaling), p-STAT3 Y705 (#9145 Cell Signaling), STAT3 (#9139 Cell Signaling), FLAG (A2220 Sigma-Aldrich), SOCS3 (ab16030 Abcam), p-ATG14 (#96752 Cell Signaling), ATG14 (#5504 Cell Signaling), GADPH (ab9484 Abcam), MAP2 (M4403 Sigma-Aldrich), β-III TUBULIN (T8578 mouse monoclonal antibody Sigma-Aldrich), p-ULK1 S758 (#6888 Cell Signaling), BECLIN 1 (sc-11427 mouse monoclonal antibody Santa Cruz Biotechnology), ACTIN (A2066 Sigma).

### Flow-cytometry

Cells were detached, washed in PBS and labelled with 10 μl of anti‐CD133‐PE (R&D System, FAB11331P) for 20 min at 4 °C. Cells were then washed twice with PBS and resuspended in 0.3 ml of 0.1% BSA/PBS and analysed by flow-cytometry.

Apoptosis analyses were performed with the Annexin V-APC kit according to the manufacturer’s protocol. Briefly, spent culture medium containing detached cells was collected, mixed with trypsinized cells and centrifuged at 300*g* for 5 min. After washing once in ice-cold 1 × PBS, cells were incubated in Annexin V Incubation reagent (1% Annexin V-APC, 1 × propidium iodide solution, in 1 × binding buffer) for 15 min in the dark.

### Xenograft mouse model for in vivo studies

Immunocompromised NSG (NOD scid gamma) mice (strain NOD.Cg-Prkdc^scid^ Il2rg^tm1Wjl^/SzJ) and CD1 athymic Nude mice were purchased from Charles River and maintained in the animal facility *Plaisant Castel Romano* (where intraperitoneal model for the bioluminescence monitoring of the tumour using IVIS Image System was performed) in Rome. All in vivo experiments were in compliance with the ethical international, EU and national requirements and were approved by the Italian Health Ministry (D.lgs 26/2014; No. 03/2013; No. 88/2016-PR; No. 1011/2020-PR). For the orthotopic in vivo model, adult female mice were anesthetized by intraperitoneal injection of ketamine (10 mg/kg) and xylazine (100 mg/kg). The posterior cranial region was shaved and placed in a stereotaxic head frame. D283-Med cells were prepared from fresh culture to ensure optimal viability. AMBRA1 was silenced as reported in Lentiviral Preparation section and cells were injected 96 h post infection. D283-Med cells (2 × 10^5^ per 3 μl) were stereotaxically implanted into the cerebellum at an infusion rate of 1 μl/min using the following coordinates, according to the atlas of Franklin and Paxinos: 6.6 mm posterior to the bregma; 1 mm lateral to the midline; and 2 mm ventral from the surface of the skull. Briefly, after injection, the cannula was kept in place for about 5 min for equilibration of pressures within the cranial vault. The skin was closed over the cranioplastic assembly using metallic clips [69]. For CQ treatment, 4 days following tumour implantation, the animals were randomly divided into two groups and CQ (60 mg/kg) or PBS was injected intraperitoneally once a day for 30 days. For the combination experiment, WP1066 was dissolved in a mixture of 20 parts dimethylsulfoxide (DMSO) to 80 parts polyethylene glycol (PEG) 300 (Sigma-Aldrich, St Louis, MO). Treatment was started 4 days after intracerebellar implantation, with doses of WP1066 (40 mg/kg) injected by oral gavage (o.g.) in a vehicle of DMSO/PEG300 (20 parts/80 parts) and CQ (60 mg/kg) every other day. Five mice per experimental group were used, including treatment with the DMSO/PEG300 vehicle alone in the control group. Tumor growth was evaluated using IVIS imaging system (PerkinElmer) once a week. Briefly, a constant region of interest was drawn over the tumour regions and the intensity of the signal measured as total photon/sec/cm^2^/sr (p/s/cm^2^/sr), as described previously [24]. After about 4 weeks, animals were sacrificed, and brains were fixed in 4% formaldehyde in 0.1 mol/l phosphate buffer (pH 7.2) and paraffin embedded. Sections were cut and stained with H&E. For IHC/IF, sodium citrate (pH 6.0) heat-mediated antigen retrieval was performed and staining was carried out using antibodies directed against: Ki67 (ab15580 Abcam), p62 (PM045 MBL), LC3 (NB100-2220 Novus Biologicals), AMBRA1 (26190002 Novus Biologicals), CD133 (#86781 Cell Signaling), SYNAPTOPHYSIN (ab 32127 Abcam), NESTIN (ab 22035 Abcam), β-III TUBULIN (T8578 Sigma-Aldrich). Slides were then mounted using Leica CV Ultra mounting medium and assessed by an experienced pathologist (F.D.C.) blinded to cell identity.

Spinal cords were decalcified with 10% EDTA (pH 7.2) for 2 weeks before embedding into paraffin blocks. Tissues were immunostained with anti-Human Nuclei (Millipore) and/or anti-Ki67 (Abcam).

### In vivo transfection and drug treatment

Mice bearing MB_Group3_ induced by c-Myc and Otx2 were generated as previously described [10]. In brief, P0 CD1 mice cerebella were transfected in vivo with the following plasmids: pPBase (encoding a hyperactive form of the piggyBac transposase), together with the piggyBac donor plasmids pPB CAG c-Myc and pPB CAG Otx2-IRES-VenusNLS. The pPB CAG Luc plasmid (encoding the firefly Luciferase) was always co-transfected as a reporter. pPBase and piggyBac donor plasmids were mixed at a 1:4 ratio. Plasmid DNA and in vivo-jetPEI transfection reagent (Polyplus-transfection) were mixed according to the manufacturer’s instructions. Newborn CD1 mice were anesthetized on ice for 2 min, placed on a stage in a stereotactic apparatus and medially injected at lambda: −3.6 D/V: −1.6 with 4 µl of transfection mix using a pulled glass capillary and a FemtoJet microinjector (Eppendorf). Mice at P18 were subjected to bioluminescence imaging of luciferase activity to monitor the effective transfection of cerebella. Animals were intraperitoneally administered 150 mg/kg d-Luciferin (Santa Cruz Biotechnology) and anesthetized with 2% isoflurane. Bioluminescent signal was captured using the In-vivo Xtreme system (Bruker). Only mice carrying luciferase signals were selected for drug treatment and divided randomly in the two treatment cohorts.

For the drug treatment, mice at P20 were intraperitoneally injected every other day for 24 days with a combination of 60 mg/kg chloroquine and 30 mg/kg WP1066, or with vehicles as control. During and after the treatment, animals were monitored for signs of morbidity and were sacrificed at a humane endpoint or at the experimental endpoint (3 months). All experiments were done with all relevant ethical regulations for animal testing and research. The experiments were approved by the Italian Ministry of Health as conforming to the relevant regulatory standards.

Survival analysis was performed calculating the lifespan in days of every mouse treated with drugs or vehicles. Mice which were sacrificed at the experimental endpoint or died due to undetermined causes during the study were censored in the analysis. Data were displayed using the Kaplan–Meier format, and statistical significance of the results was tested using the Log-rank (Mantel–Cox) test.

### Statistical analyses

Statistical analysis was performed using GraphPad Prism software. All data are presented as means ± SEM unless stated otherwise. Comparisons between different groups were made using Student’s *t*-test, ANOVA or non-parametric ANOVA as appropriate and as indicated in the figure legends.

The correlation analysis was carried out through R2 (Genomics Analysis and Visualisation Platform; http://r2.amc.nl). The protein densitometry for western blotting quantification used was carried out using the ImageJ software to calculate the relative and normalized densities of peaks corresponding to the bands for proteins of interest, and those relative to the loading-control bands. The statistical significance of Kaplan–Meier survival curves was assessed using the log-rank (Mantel-Cox) test. *p* values of 0.05 or lower were considered statistically significant for all experiments.

## Results

### AMBRA1 is differentially expressed in MB subtypes and its levels correlate with patient survival

AMBRA1 is known to be crucial for both nervous system development and autophagy. Indeed, in mice at embryonic day (E)8.5, strong AMBRA1 staining is detected throughout the neuroepithelium and, later in development, it becomes abundant in the entire developing nervous system, as well as in other tissues [29, 50, 58]. Indeed, in adult mice cerebellum, Ambra1 expression depends on the neuronal subtype: for instance, in the cerebellar cortex, Ambra1 is highly expressed in the Purkinje cell layer, whereas it is almost excluded from the granule cell layer [83]. Interestingly, by analysing Ambra1 protein levels in various neural stem cell types, we found a positive expression in E14.5 neuronal stem cells (NSCs), in cerebellar NSCs (cNSCs) as well as in granule neuron precursors (GNPs) from postnatal P4 mice (Supp. Fig. 1a, b, online resource). Altogether, these results confirm the prominent role of AMBRA1 in early nervous system formation and highlight its importance in cerebellar development.

Genetic programs involved in early development of the nervous system are often hijacked in brain cancers. To investigate the role of AMBRA1 in MB, we collected tumour samples of 47 cases (Supp. Table S1), including classic (*n* = 30), desmoplastic/nodular (DN) (*n* = 7), and LCA (*n* = 10) MB, and AMBRA1 expression was evaluated by immunohistochemistry (IHC). As shown in Fig. 1a, b, AMBRA1 is abundantly expressed in both classic and LCA subtypes, when compared with DN. Interestingly, when we examined MB samples in a genomic subgroup-specific fashion, we found that AMBRA1 expression is much higher in MB_WNT_ and MB_Group3_ than in the other two groups (Fig. 1c). Real-time PCR (qPCR) in patient-derived samples confirmed that *AMBRA1* mRNA levels are increased in MB_WNT_ and, at a less extent, in the more heterogeneous MB_Group3_ tumors (Suppl. Fig. 1c, online resource). To further validate the pattern of AMBRA1 expression observed in MB subgroups, we analysed samples from two public MB datasets (deposited at http://r2.amc.nl; Pfister *n* = 167 samples and Cavalli *n* = 763 samples) [13, 48, 64]. Consistent with our data, we found that *AMBRA1* transcriptional levels are significantly higher in both MB_WNT_ and MB_Group3_ compared to both MB_SHH_ and MB_Group4_ (Fig. 1d) (Supp. Fig. 1d, online resource).

In the light of the recent recognized heterogeneity across Group 3 and 4, we found that *AMBRA1* is significantly upregulated in both G3α and G3β compared with other MB subtypes (SHHα,γ,δ–G4α,β) (Fig. 1e). Moreover, we decided to inspect the eight molecular subtypes of MB_Group3_ and MB_Group4_ described by Northcott et al*.* [64], that are highly congruent with the eight groups reported by Sharma et al. [39, 84]. As reported in Supp. Fig. 1e (online resource), we observed that *AMBRA1* is significantly more expressed in subtype III (a *bona fide* Group3 subtype) and MB_Group3_ than in MB_Group3/4_ and MB_Group4._

Of the highest importance in MB studies, and given these findings, we decided to stratify our cohort of MB patients into two groups according to AMBRA1 immunostaining intensities (negative/weak expression *versus* median/strong positive). Intriguingly, patients with higher AMBRA1 levels (*n* = 27) have poorer survival than those with lower AMBRA1 expression (*n* = 19, *p* = 0.01; Fig. 1f). This result is fully in line with what we found by analysing the overall survival of MB_Group3_ patients listed in the Cavalli dataset (*n* = 113; *p* = 0.026), and even more significant when analysing G3α subtype (*n* = 55; *p* = 0.010) (Fig. 1g,h).

Of note, it is known that MYC family members play a critical role in each of the MB subgroups [78]. c-MYC expression was found to be very high in both MB_WNT_ and MB_Group3_ (Supp. Fig. 1f, online resource) [13, 39, 64]. However, MB_WNT_ and MB_Group3_ are characterized by a completely different overall survival, and when enhancing c-MYC expression in mouse models of MB, none of these results in the formation of WNT subgroup [78]. MB_WNT_ patients, indeed, are characterized by excellent prognosis, which may be partly due to alterations in the brain vasculature that could increase chemotherapy penetration [67].

Interestingly, despite the highly heterogeneous nature, at both molecular and histological levels, of the samples collected in public databases, we found a moderately positive correlation between *AMBRA1* and *c-MYC* across the entire Cavalli dataset (*r* = 0.316, *p* = 3.7 × 10^–19^) and subtypes I-VIII (merged analysis of Group 3 and Group 4) described by Northcott et al. (*r* = 0.338, *p* = 4.23 × 10^–4^) (Supp. Fig. 1 g, h, online resource). Intriguingly, in a combined evaluation within this recent MB subclassification, *AMBRA1* and *c-MYC* levels highly correlate among them in Group 3/4 (subtypes I, V, VII; *r* = 586, *p* = 6.71 × 10^–4^) (Supp. Fig. 1i, online resource). Further, although univariate survival analyses display significantly shorter progression-free survival associated with both high *AMBRA1* and c-*MYC* levels in MB_Group3_ and even in G3α (Cavalli dataset), multivariate Cox modelling shows that both *AMBRA1* and *c-MYC* are independent risk factors for progression-free survival (see Supp. Fig. 1j, online resource), in both MB_Group3_ and G3α subtype. Additionally, by analysing *AMBRA1* levels in non-amplified c-MYC *versus* amplified c-MYC patients (G3γ-Cavalli dataset), no differences in AMBRA1 levels are found (Supp. Fig. 1k, online resource).

Next, *AMBRA1* levels were investigated among normal cerebellum (NC) and different MB cell lines belonging to different MB subgroups and having distinctive c-MYC levels: DAOY = MB_SHH low c-MYC_, D283-Med = MB_Group3 high c-MYC,_ CHLA-01-Med = MB_Group3 c-MYC amplified_ [44, 90]. In agreement with our data on MB patients, we found that *AMBRA1 mRNA* levels were higher in both D283-Med and CHLA-01-Med (Fig. 1i).

These findings prompted us to look for a molecular correlation between AMBRA1 and c-MYC expression in MB.

### AMBRA1 expression in MB_Group3_ depends on c-MYC interaction with MIZ-1

**Fig. 2:**
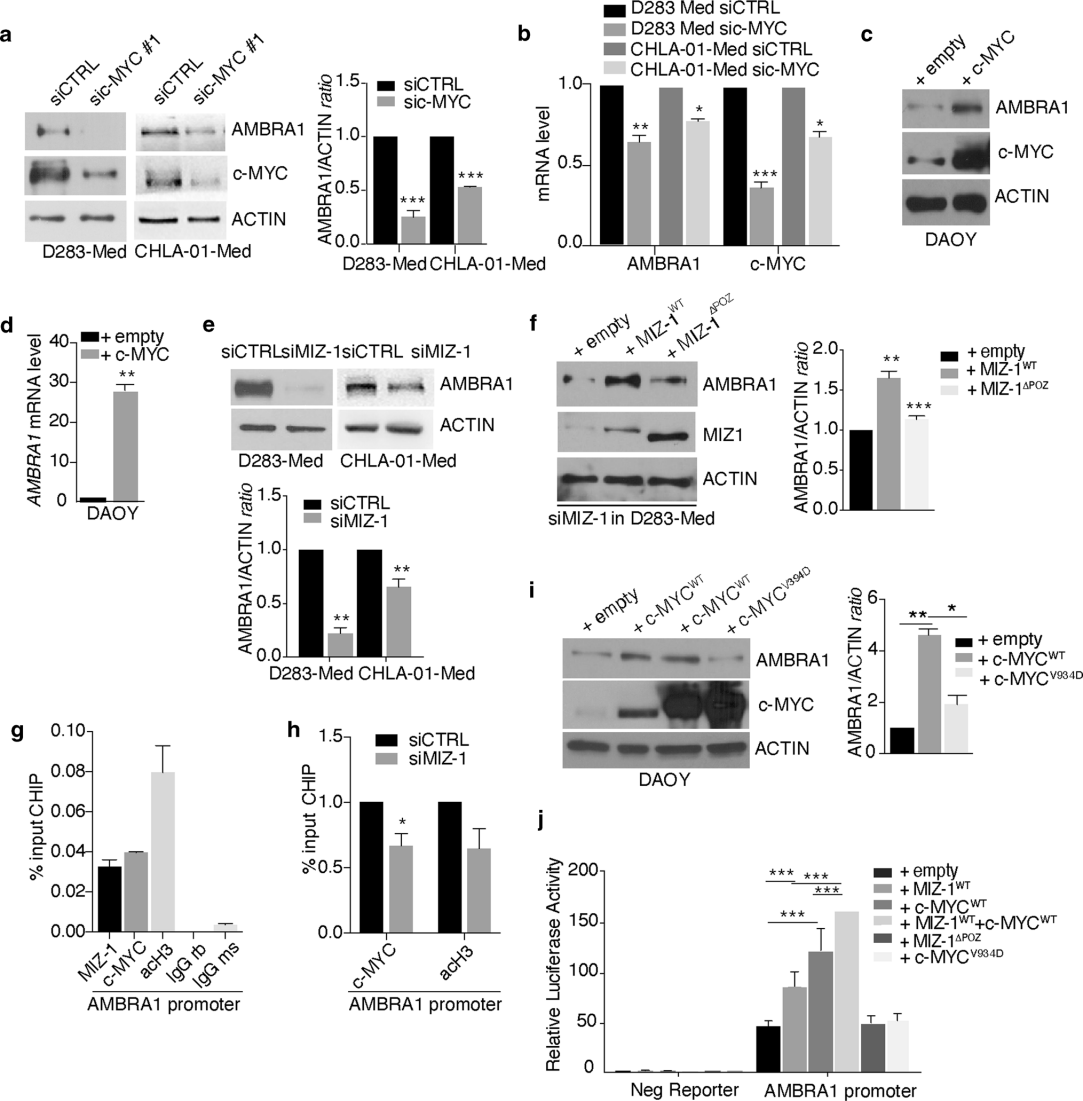
c-MYC/MIZ-1 complex regulates AMBRA1 expression in MB. **a** c-MYC expression was downregulated in both D283-Med and CHLA-01-Med cells using specific RNAi oligonucleotides (sic-MYC) or unrelated oligos as negative control (siCTRL). Levels of AMBRA1, c-MYC and ACTIN were analysed by WB. Densitometric analysis of AMBRA1 levels over ACTIN is also shown (right panel). siCTRL was arbitrarily defined as 1.00. Data are expressed as the mean ± SEM (*n* = 3) and analysed by unpaired Student's *t*-test (***p < 0.001). **b** D283-Med and CHLA-01-Med cells were treated as in (**a**). Both *AMBRA1* and c-*MYC* mRNA levels were analysed by qPCR. *GADPH* and *B2M* were used as internal control. Data are expressed as the mean value ± SEM (*n* = 3). siCTRL was arbitrarily defined as 1.00. Data were analysed by unpaired Student's *t*-test (*p < 0.05, **p < 0.01, ***p < 0.001). **c** DAOY cells were transfected with empty or c-MYC encoding vectors, respectively; levels of AMBRA1, c-MYC and ACTIN were detected by WB, respectively. **d** DAOY cells were treated as in (**c**). *AMBRA1* mRNA levels were analysed by qPCR. *GADPH* and *B2M* were used as internal control. Data are expressed as the mean value ± SEM (*n* = 3). Data were analysed by unpaired Student's *t*-test (***p < 0.01). **e** MIZ-1 expression was downregulated in both D283-Med and CHLA-01-Med cells using specific RNAi oligonucleotides (siMIZ-1) or unrelated oligos as negative control (siCTRL). Levels of AMBRA1 and ACTIN were analysed by WB. Densitometric analysis of AMBRA1 levels over ACTIN is also shown (right panel). siCTRL was arbitrarily defined as 1.00. Data are expressed as the mean ± SEM (*n* = 3) and analysed by unpaired Student's *t*-test (***p < 0.01). **f** D283-cells were treated ad in (**e**). Then, some of them were transfected with empty, MIZ-1^WT^ or MIZ-1^ΔPOZ^ plasmids respectively. Levels of AMBRA1, MIZ-1 and ACTIN were analysed by WB. Densitometric analysis of AMBRA1 levels over ACTIN is also shown (right panel). Data are expressed as the mean ± SEM (*n* = 3) and analysed by one-way analysis of variance (ANOVA) followed by Tukey post hoc test (**p < 0.01; ***p < 0.001). **g** Binding of both MIZ-1 and c-MYC to AMBRA1 promoter. Data are expressed as the mean value ± SEM (*n* = 2). acH3 = acetyl histone H3; IgG rb = rabbit; IgG ms = mouse. **h** Binding of c-MYC (and acH3) to AMBRA1 promoter in MIZ-1-depleted cells. Data are expressed as the mean value ± SEM (*n* = 3). siCTRL was arbitrarily defined as 1.00 and analysed by unpaired Student's *t*-test (*p < 0.05). **i** DAOY cells were transfected with empty, c-MYC^WT^ (two different constructs) and c-MYC^V394D^ plasmids, respectively. Levels of AMBRA1, c-MYC and ACTIN were analysed by WB. Densitometric analysis of AMBRA1 levels over ACTIN is also shown (right panel). Data are expressed as the mean ± SEM (*n* = 3) and analysed by one-way analysis of variance (ANOVA) followed by Tukey post hoc test (*p < 0.05; **p < 0.01). **j** HEK293 cells were transiently transfected with plasmids containing promoter sequences of *AMBRA1* in front of Gaussia luciferase construct and with expression vectors encoding Miz-1^WT^, Miz-1^ΔPOZ^, c-MYC^WT^ and c-MYC^V394D^ respectively. Empty vectors were used as control. Cells were transfected for 2 days and supernatant were collected for the luciferase assays. Luciferase activity was normalized to secreted alkaline phosphatase. Data are presented as the mean ± SEM (*n* = 3) and analysed by two-way analysis of variance (ANOVA) followed by Tukey post hoc test (***p < 0.001)

To test the interplay between c-MYC and AMBRA1, we silenced c-MYC in both D283-Med and CHLA-01-Med cells. Upon c*-MYC* downregulation (for siRNA efficacy see Supp. Fig. 2a, online resource), we found that, in *c-MYC*-downregulated cells, AMBRA1 decreases at both mRNA and protein levels (Fig. 2a,b). Conversely, when we exogenously expressed c-MYC in DAOY cells, we observed a significant upregulation of AMBRA1 (Fig. 2c,d), this result supported a role for c-MYC in regulating AMBRA1 expression. To further investigate how c-MYC controls AMBRA1 levels, we next analysed the involvement of MIZ-1, a c-MYC cofactor known to directly regulate AMBRA1 transcription [98, 99] in both D283-Med and CHLA-01-Med cells. Similarly to c-MYC, when we down-regulated *MIZ-1* by RNA interference (after selection of the most efficient anti-MIZ-1 siRNAs), we observed a significant decrease of AMBRA1 at both mRNA and protein levels (Fig. 2e) (Supp. Fig. 2b, online resource). By contrast, *MIZ-1* overexpression was not able per se to increase AMBRA1 protein levels (Supp. Fig. 2c, online resource), this impinging on the c-MYC-dependence of MIZ-1 regulatory effect on AMBRA1. Given the crucial role of the Pox virus and Zinc finger (POZ) domain for the transactivation and repression activity of MIZ-1, to assess whether MIZ-1 transcription activity is necessary to control *AMBRA1* expression, we performed rescue experiments in MIZ-1-silenced cells. As shown in Fig. 2f and Supp. Fig. 2d (online resource), we found that rescue with full length MIZ-1 (MIZ-1^WT^) but not with POZ-depleted MIZ-1 (MIZ-1^∆POZ^) restored AMBRA1 protein levels, this implying the requirement of MIZ-1 activity to *AMBRA1* transcriptional upregulation. Given that c-MYC and MIZ-1 are able to act as a complex and that they represent a hallmark in MB_Group3_ [95], we examined whether c-MYC-MIZ-1 interaction was necessary for AMBRA1 upregulation. First, by chromatin immunoprecipitation (ChIP) experiments, we found that both c-MYC and MIZ-1 bind the *AMBRA1* promoter region (Fig. 2g), although c-MYC recruitment depends epistatically on the presence of MIZ-1 (Fig. 2h). To better investigate the relationship among these two transcriptional factors in AMBRA1 regulation, we used a specific c-MYC mutant construct (c-MYC^V394D^) deficient for MIZ-1 binding [98]. As reported in Fig. 2i, exogenous expression of c-MYC^WT^ increases AMBRA1 protein levels, at variance with that of c-MYC^V394D^, supporting the idea that c-MYC-MIZ-1 association is necessary for AMBRA1 regulation. Finally, to test whether or not MIZ-1/c-MYC complex activated *AMBRA1* transcription, we engineered a fragment of the *AMBRA1* promoter encompassing the MIZ-1-binding site [95], and performed transient reporter assays. Expression of MIZ-1 and c-MYC alone stimulates expression of the *AMBRA1*-promoter reporter gene, whereas Miz-1^∆POZ^ and c-MYC^V394D^ are ineffective in its transactivation (Fig. 2j). Interestingly, co-expression of both MIZ-1 and c-MYC significantly increases *AMBRA1* promoter efficacy, relative to both single transfections and control vector. In sum, these data demonstrate that c-MYC-MIZ-1 interaction is required for the regulation of AMBRA1 expression in MB_Group3_ stem cells.

### AMBRA1 strictly controls stem cell potential in various MB-derived cell lines

**Fig. 3:**
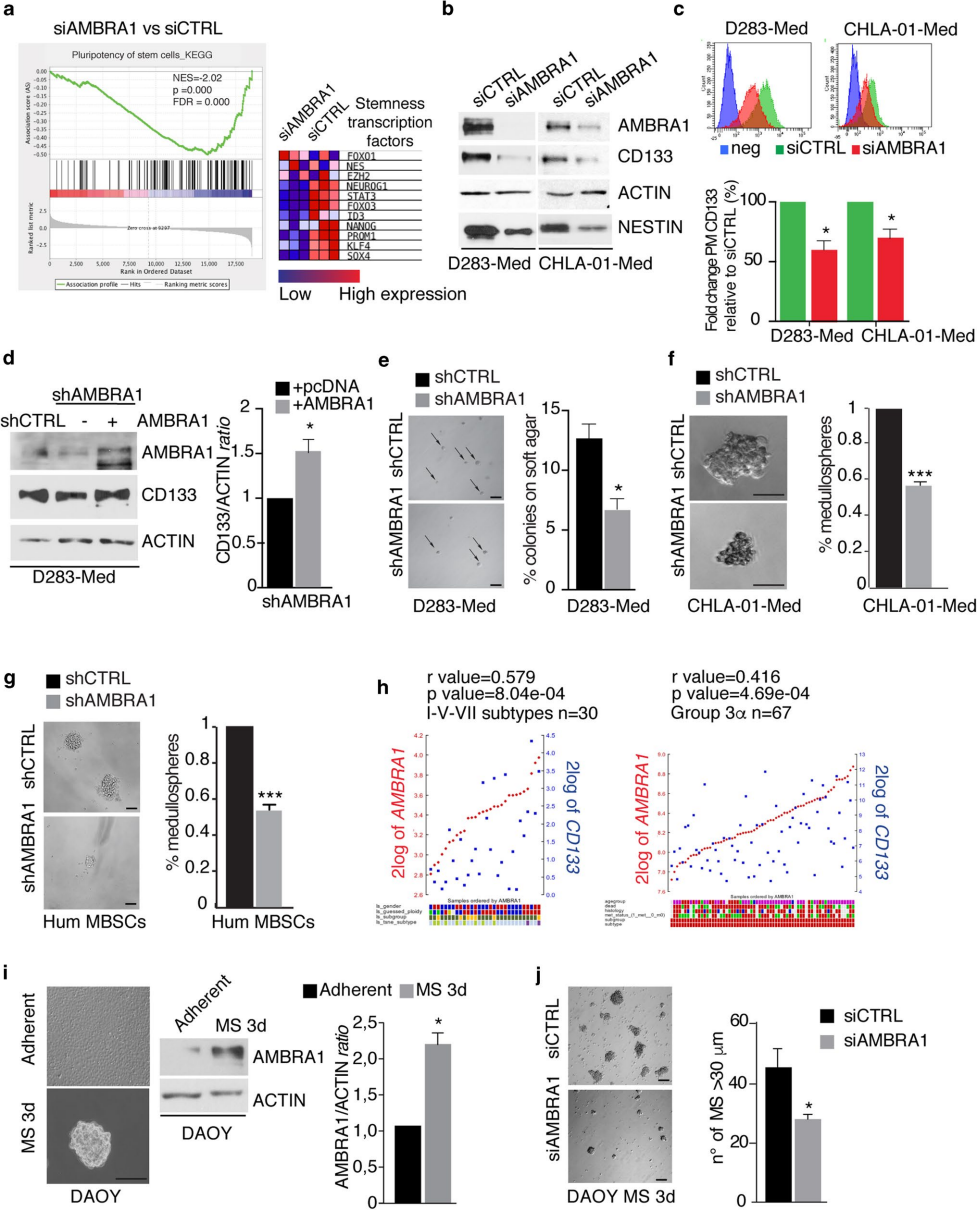
Inhibition of AMBRA1 impairs stem potential in various MB cell lines. **a** GSAA enrichment plot showing that loss of AMBRA1 in D283-Med cells (*n* = 3 independent experiments) results in downregulation of the pluripotency of stem cell pathway. Right, a heatmap showing the expression of representative stem-related genes in D283-Med cells with (siAMBRA1) or without (siCTRL) AMBRA1 knockdown. Data were generated from RNA-seq analyses. **b** AMBRA1 expression was downregulated in both D283-Med and CHLA-Med-01 cells using specific RNAi oligonucleotides (siAMBRA1) or unrelated oligos as negative control (siCTRL). Levels of AMBRA1, CD133, NESTIN and ACTIN were analysed by WB. **c** D283-Med and CHLA-Med-01 cells were treated as in (**b**). The percentage of plasma membrane (PM) CD133 positive cells was assessed by flow-cytometry. Representative histograms of CD133 mean of fluorescence intensity (MFI) in both D283-Med and CHLA-Med-01 cells are shown. Data are expressed as the mean value ± SEM (*n* = 3). Data were analysed by unpaired Student's *t*-test (**p < 0.05). **d** AMBRA1 expression was downregulated by lentiviral infection (shAMBRA1) or negative control (shCTRL). Then, some of them were transfected with empty or AMBRA1-encoding plasmids, respectively. Levels of AMBRA1, CD133, and ACTIN were analysed by WB. Densitometric analysis of CD133 levels over ACTIN is also shown (right panel). Data are expressed as the mean ± SEM (*n* = 3) and analysed by unpaired Student's *t*-test (*p < 0.05). **e** AMBRA1 was downregulated in D283-Med cells by RNAi. After 24 h, cells were plated on double-layer agar and the number of colonies was assessed after 7 days. Representative images (4 ×) (after 7 days of culture) are shown. Data are expressed as the mean value ± SEM (*n* = 3). Data are analysed by unpaired Student's *t*-test (*p < 0.05). **f**, **g** Self-renewal ability (percentage of medullospheres) of both CHLA-01-Med cells and primary human MBSCs after AMBRA1 lentiviral downregulation (shAMBRA1). Data are expressed as the mean ± SEM (*n* = 3) and analysed by unpaired Student's *t*-test (*p < 0.05). Representative images 40 × scale bar 50 µm  (**f**) and 20 × scale bar 50 µm (**g**) are shown. **h** Gene expression correlation analyses between transcripts for *AMBRA1* and *CD133* in MB samples from both the Pfister (I–V–VII subtypes *n* = 30) and the Cavalli datasets (G3α *n* = 67), respectively. Statistically significant positive correlations are shown (*r* = 0.570 *p* = 8.04e−04, *r* = 0.416 *p* = 4.69e−04). **i** DAOY cells were cultured as medullospheres for 3 days (3d). Representative images (40 ×) are shown. Scale bar: 50 μm. AMBRA1 and ACTIN levels were analysed by WB. Densitometric analyses of AMBRA1 over ACTIN is also shown (bottom panel). Data are expressed as the mean ± SEM (*n* = 3) and analysed by unpaired Student's *t*-test (*p < 0.05). **j** AMBRA1 expression was downregulated in DAOY cells using specific RNAi oligonucleotides (siAMBRA1) or unrelated oligos as negative control (siCTRL). After 24 h, cells were cultured as medullospheres. Representative images (20 ×) were shown. Amount of medullospheres generated (diameter > 30 μm) were analysed after 3 days of culture. Data are expressed as the mean ± SEM (*n* = 3) and analysed by unpaired Student's *t*-test (*p < 0.05)

Given the differential AMBRA1 expression in MB subgroups, among which MB_Group3_ is the most aggressive, we decided to check for the presence of an AMBRA1-dependent phenotype. D283-Med cells were selected as the best cell model to represent MB_Group3_ in this study of the mechanism of action of AMBRA1, because of higher AMBRA1 levels compared to CHLA-01-Med cells. To this aim, we first performed an RNA-seq analysis, characterizing the global gene expression changes induced by *AMBRA1* siRNA-mediated downregulation. Indeed, using gene set enrichment analysis (GSAA), we observed that AMBRA1 loss reduces primarily the expression of gene sets that have been linked to represent hallmarks of stemness properties of cerebellar neuronal stem cells and MB stem cells (MBSCs) [59, 72] (Fig. 3a) (Supp. Fig. 3a, online resource). Subsequently, based on this finding and on the knowledge that MB_Group3_ cells display features of partially committed neural stem and progenitor cells [36, 47], we checked for the expression of the CD133 and NESTIN stem-cell markers among MB cell lines, and detected higher expression in c-MYC^+^ cells (D283-Med and CHLA-01-Med), when compared with DAOY (Supp. Fig. 3b,c, online resource). Of the highest importance, depletion of *AMBRA1* by siRNAs strongly reduces expression of stem-cell markers at both mRNA and protein levels (Fig. 3b-c) (Supp. Fig. 3d, online resource). Of note, when we reintroduced *AMBRA1* in stably AMBRA1-interfered (shAMBRA1) D283-Med cells, we were able to rescue CD133 protein levels, with this implying AMBRA1 requirement for CD133 upregulation (Fig. 3d).

Then, to further investigate the effects of AMBRA1 modulation on MB cell clonogenicity, we performed a soft-agar assay for D283-Med cells and a clonogenic assay for CHLA-01-Med cells. As shown in Fig. 3e,f, we found that loss of *AMBRA1* results into a significant reduction in clonogenic ability [measured as the number of medullospheres (MS) formation] of all MB cells studied. To strengthen our findings, we also used MBSCs from human MB (hum MBSCs) [1], confirming a significant decrease of both their clonogenic potential and stemness marker expression levels after AMBRA1 silencing (Fig. 3g) (Supp. Fig. 3e, online resource). Then, to investigate the correlation between AMBRA1 expression and patient stem cell potential, we analysed (i) CD133 levels in 43 MB primary samples previously analysed for AMBRA1 by IHC, and (ii) *CD133* expression in correlation with *AMBRA1* in both Cavalli and Pfister datasets, by looking at different subtypes. Indeed, 18/19 samples highly expressing AMBRA1 are also positive for CD133 (Supp. Fig. 3f, online resource); moreover, we found that *AMBRA1* and *CD133* highly and significantly correlate in Group 3/4 (subtypes I–V–VII Pfister dataset; *r* = 579, *p* = 8.94 × 10^–4^) and in G3α (Cavalli dataset, *r* = 416 *p* = 4.69 × 10^–4^) (Fig. 3h) but not in the other subtypes analysed (G3β, G3γ, II–III–IV and VI–VIII subtypes) (Supp. Fig. 3g,h, online resource). We finally confirmed our results by culturing DAOY cells that usually express low levels of stemness markers in stem-cell media. Indeed, they give rise to MS and upregulate stem markers, including *SOX2* and *OCT4* (Supp. Fig. 3i, online resource) in addition to what previously described for c-*MYC, NESTIN, NANOG, CD133* [2]*.* Interestingly, we could detect, in this context, an increase in *AMBRA1* expression (Fig. 3i). By contrast, knockdown of *AMBRA1* strongly inhibits the ability of DAOY cells to form MS, which also resulted to be significantly smaller in size (Fig. 3j), as well as the expression of stemness markers (Supp. Fig. 3j, online resource). In agreement with previous results, using retinoic acid to induce D283-Med cells differentiation, we were able to induce a decrease in AMBRA1 expression (Supp. Fig. 3 k,l, online resource), this supporting the evidence of a close link between AMBRA1 and MB stem cell potential.

### Autophagy enhances stemness in MB cells in vitro

**Fig. 4:**
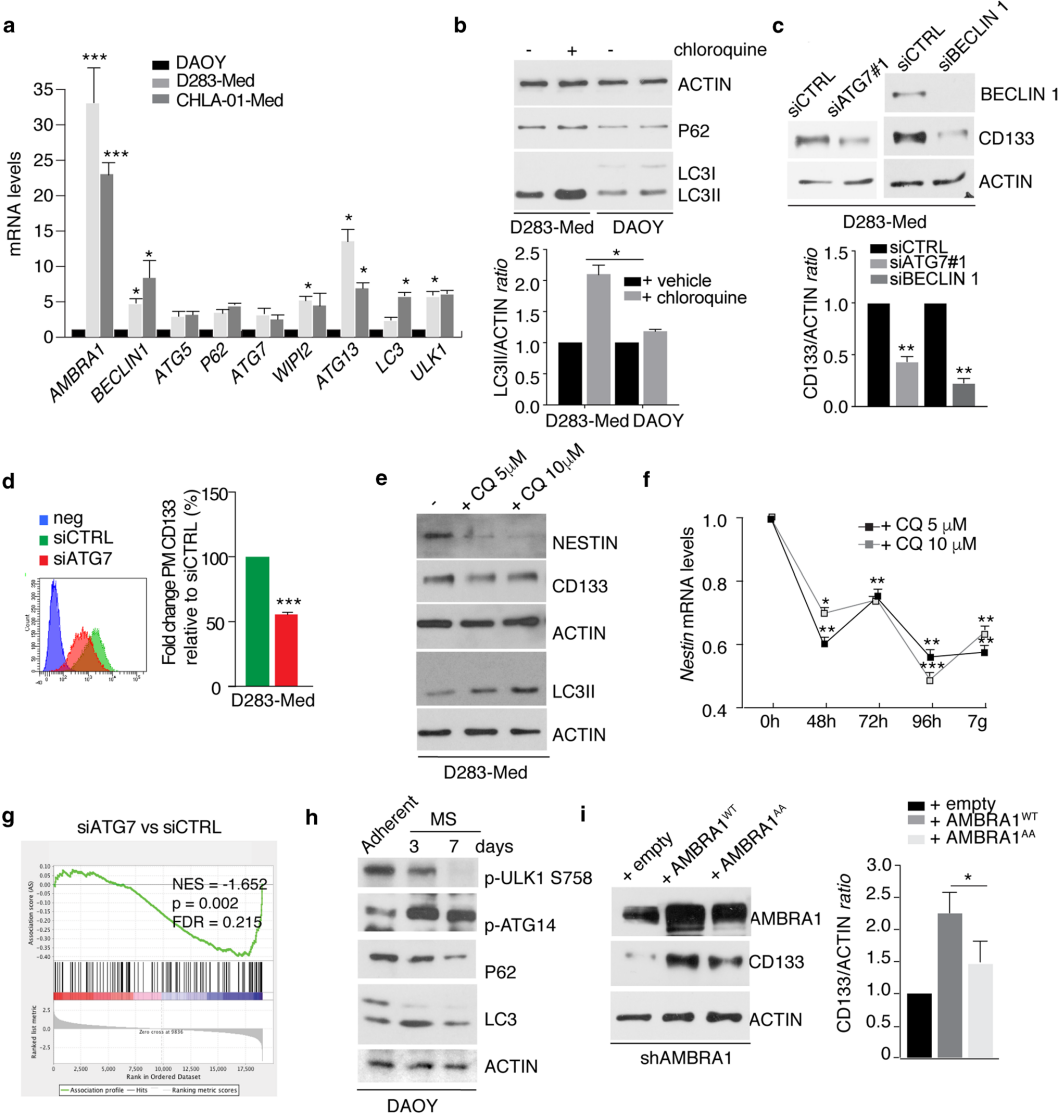
Autophagy enhances stemness in MB. **a** qPCR analysis of autophagy-related genes mRNA in different MB cell lines. *GADPH* and *B2M* were used as internal control. DAOY cells were arbitrarily defined as 1.00. Data are expressed as the mean value ± SEM (*n* = 6). Data were analysed by one-way analysis of variance (ANOVA) followed by Tukey post hoc test. (*p < 0.05; ***p < 0.001). **b** Both D283-Med and DAOY cells were treated with CQ (20 μM) for 1 h. Levels of LC3, P62, and ACTIN were analysed by WB. Densitometric analysis of LC3 II levels over ACTIN is also shown. Data are expressed as the mean ± SEM (*n* = 3) and analysed by one-way analysis of variance (ANOVA) followed by Tukey post hoc test (**p < 0.01). **c** Both *ATG7* and *BECLIN 1* expression was downregulated in D283-Med cells using specific RNAi oligonucleotides (siATG7 or siBECLIN 1, respectively) or unrelated oligos as negative control (siCTRL). Levels of CD133, BECLIN 1 and ACTIN were analysed by WB. Densitometric analysis of CD133 levels over ACTIN is also shown. Data are expressed as the mean ± SEM (*n* = 3) and analysed by unpaired Student's *t*-test (**p < 0.01). The downregulation of ATG7 was analysed by qPCR. **d** D283-Med cells were treated as in (**c**). The percentage of plasma membrane (PM) CD133 positive cells was assessed by flow-cytometry. Representative histograms of CD133 mean of fluorescence intensity (MFI) are shown. Data are expressed as the mean value ± SEM (*n* = 3). Data were analysed by unpaired Student's *t*-test (**p < 0.01). **e** D283-Med cells were treated with two different concentration of CQ (5 and 10 μM, respectively) for 48 h. Levels of CD133, NESTIN, LC3 and ACTIN were analysed by WB. **f** D283-Med cells were treated as in (**e**) for different time points. *NESTIN* mRNA levels were analysed by qPCR. *GADPH* and *B2M* were used as internal control. Data are expressed as the mean value ± SEM (*n* = 3). Data were analysed by one-way analysis of variance (ANOVA) followed by Tukey’s post-hoc test. (*p < 0.05, **p < 0.01). **g** GSAA enrichment plot showing that loss of ATG7 in D283-Med cells (3 independent experiments) results in downregulation of the pluripotency of stem cell pathway. Data are generated from RNA-seq analyses. **h** DAOY cells were cultured as medullospheres for 3 and 7 days. Levels of p-ULK1 758, p-ATG14, LC3, P62 and ACTIN were analysed by WB. **i** AMBRA1-silenced D283-Med cells (shAMBRA1) were transfected with empty, AMBRA1^WT^ and AMBRA1^AA^ mutant constructs, respectively. Levels of AMBRA1, CD133 and ACTIN were analysed by WB. Densitometric analysis of CD133 levels over ACTIN is also shown (right panel). Data are expressed as the mean ± SEM (*n* = 3) and analysed by one-way analysis of variance (ANOVA) followed by Tukey post hoc test. (*p < 0.05)

Given the key roles of AMBRA1 in the upstream regulation of autophagy [18, 19, 63, 92], we decided to study the role of this process in MB stemness. First, we analysed the expression levels of autophagy regulators, such as *BECLIN 1, ATG5, P62, ULK1, LC3, ATG7, ATG13* and *WIPI2* among MB cell lines (Fig. 4a). Among the genes known to regulate autophagy, many of them are more expressed in both D283-Med and CHLA-01-Med than in DAOY cells, with AMBRA1 showing the most evident and consistent differential expression. Then, to determine whether MB_Group3_ cells also exhibited increased autophagy activity, we used chloroquine (CQ: an inhibitor able to block late stages of autophagy, commonly used also to assess the autophagy flux) on both D283-Med and DAOY cell lines. As shown in Fig. 4b, we detected an increased lipidation of the autophagy marker LC3 in D283-Med than in DAOY cells, suggesting an increased basal autophagy. In addition, when two autophagy-related factors (*BECLIN 1* and *ATG7*) were silenced in D283-Med cells (see Supp. Fig. 4a online resource for siRNA selection), a clear decrease in the stem phenotype was recapitulated (Fig. 4c,d) (Supp. Fig. 4a, online resource). Interestingly, by both acute (single treatment for 48 h) (Fig. 4e) or chronic (multiple treatments every 48 h) (Fig. 4f) administration of CQ to D283-Med cells, we obtained a decrease in the expression of stem cell markers, this implying a key role for autophagy in the maintenance of a stem-like phenotype. Consistent with this hypothesis, analysis of RNA-seq in D283-Med *ATG7*-silenced cells show that loss of *ATG7* is sufficient to decrease the expression of genes involved in stemness (Fig. 4g) (Supp. Fig. 4b,c, online resource). To extend these findings to D283-Med cells, autophagy was also measured in DAOY cells after MS induction and we found that autophagy is induced when DAOY cells are cultured as MS (Fig. 4h). Last, to dissect whether the AMBRA1 pro-autophagic activity was essential for the regulation of stem potential, we took advantage of an *AMBRA1* mutant construct (AMBRA1^AA^) impaired in its autophagic function [63]. Indeed, as illustrated in Fig. 4i, AMBRA1^AA^ is able to rescue at least in part CD133 protein levels.

Altogether, these data demonstrate that autophagy positively regulates stemness in MB cells and that pharmacological inhibition of autophagy reduces MB stem cell potential in vitro*.*

### AMBRA1 downregulation or autophagy inhibition affect MB cell survival and migration, and induce their differentiation

**Fig. 5:**
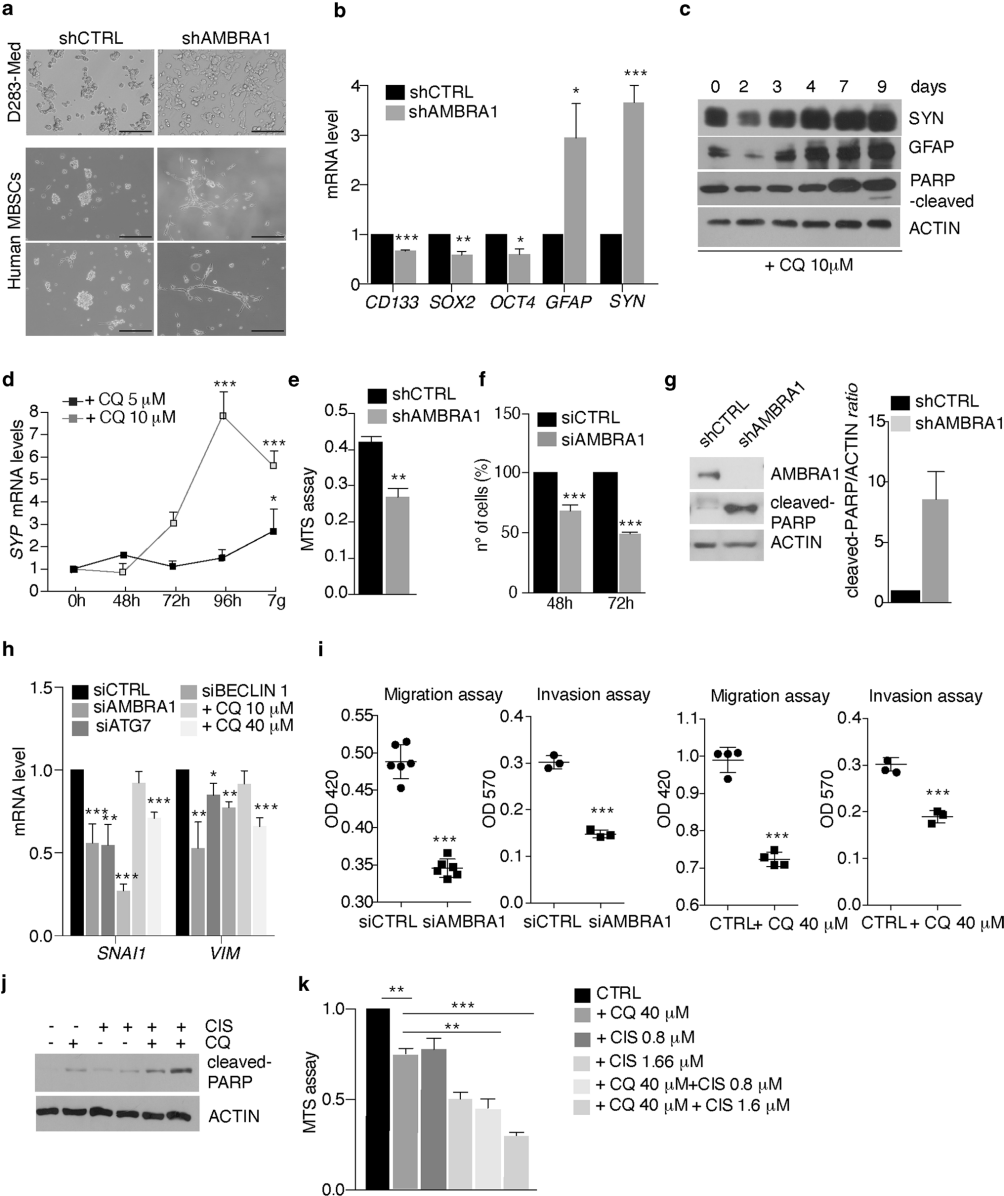
AMBRA1 downregulation or autophagy inhibition affects MB cell survival and migration, and induce cell differentiation. **a** AMBRA1 expression was downregulated by lentiviral infections (shAMBRA1) or negative control (shRNA CTRL) for 10 days. Representative images of morphological changes of both D283-Med cells and human MBSCs after AMBRA1 silencing (shAMBRA1) are shown. Scale bar: 50 μm. **b** qPCR analysis of *CD133*, *SOX2*, *OCT4*, *GFAP* and *SYNAPTOPHYSIN* (SYP) was performed in D283-Med cells after AMBRA1 silencing (shAMBRA1). *GADPH* and *B2M* were used as internal control. Data are expressed as the mean value ± SEM (*n* = 3). Data were analysed by unpaired Student's *t*-test (*p < 0.05, **p < 0.01, ***p < 0.001). **c** D283-Med cells were treated with CQ (10 μM) for different time points. SYP, GFAP, cleaved PARP and ACTIN were analysed by WB. **d** D283-Med cells were treated with two different amounts of CQ (5 and 10 μM) respectively for 7 days. Expression of *SYP* mRNA was detected by qPCR. Data are expressed as the mean value ± SEM (*n* = 3). Data were analysed by one-way analysis of variance (ANOVA) followed by Tukey post hoc test. (*p < 0.05; **p < 0.01). **e** D283-Med cells were treated as in (**a**). Proliferation was assessed by MTS assay. Data are expressed as the mean ± SEM (*n* = 3) and analysed by unpaired Student's *t*-test (**p < 0.01). **f** AMBRA1 expression was downregulated using specific RNAi oligonucleotides (siAMBRA1) or unrelated oligos as negative control (siCTRL). Proliferation was assessed by cell counting after both 48 and 72 h. Data are expressed as the mean ± SEM (*n* = 3) and analysed by unpaired Student's *t*-test (***p < 0.001). **g** D283-Med cells were treated as in (**a**). AMBRA1, cleaved-PARP and ACTIN were analysed by WB. Densitometric analysis of cleaved-PARP over ACTIN is also shown. Data are expressed as the mean ± SEM (*n* = 3) and analysed by unpaired Student's *t*-test (*p < 0.05). **h** AMBRA1, ATG7, BECLIN 1 expression was individually downregulated in D283-Med cells using specific RNAi oligonucleotides or unrelated oligos as negative control. Autophagy is also inhibited using CQ (10 and 40 μM, respectively). qPCR analysis of *SNAI1* and *VIMENTIN* (VIM) were analysed. Both GADPH and B2M were used as internal control. Data are expressed as the mean value ± SEM (*n* = 3). Data were analysed by unpaired Student's *t*-test (*p < 0.05, *p* < 0.01, ***p < 0.001). **i** D283-Med cells were treated as in (**h**) and then plated for both migration and invasion assays. Data are expressed as the mean value ± SEM (*n* = 5). Data were analysed by unpaired Student's *t*-test (***p < 0.001). **j** D283-Med cells were treated with either CQ (40 μM) or cisplatin (CIS 0.8–1.66 μM) or in combination for 72 h, respectively. Cleaved-PARP and ACTIN were analysed by WB. **k** D283-Med cells were treated as in (**j**). MTS proliferation assay was performed. Data are expressed as the mean ± SEM (*n* = 3) and analysed by one-way analysis of variance (ANOVA) followed by Tukey post hoc test (**p < 0.01, ***p < 0.001)

Given the strong effect of AMBRA1 loss on stemness, we decided to investigate whether AMBRA1 downregulation could lead to MBSCs differentiation. As a proof of concept, we cultured shAMBRA1 D283-Med cells and human primary MBSCs [72] for 10 days and analysed the expression of differentiation markers such as the Glial Fibrillary Acidic Protein (*GFAP*) and Synaptophysin *(SYP).* As reported in Fig. 5a, shAMBRA1 cells show typical morphological changes of differentiated cells in both D283-Med and human primary MBSCs, together with significant upregulation of the two differentiation markers analysed (Fig. 5b). Similarly, chronic CQ treatment led to increase of both *SYP* and *GFAP* levels in D283-Med, suggesting that also autophagy inhibition can trigger MBSCs differentiation (Fig. 5c, d).

Next, we investigated the role of AMBRA1 on MB cell proliferation. In *AMBRA1*-silenced D283-Med cells, we found a significant decrease of cell viability and cell number (Fig. 5e, f) and an increase in cell death (Fig. 5g). By contrast, no significant differences were found upon *ATG7* silencing (Supp. Fig. 5a, online resource).

Interestingly, through RNA-seq analyses performed following *AMBRA1* silencing, we found that loss of AMBRA1 induces gene sets representing inactivation of TGF-β (Supp. Fig. 5b, online resource). While previous reports have associated the TGF-β pathway with pro-migratory potential of MB [101], a strict correlation between stemness and motility has been found to be crucial for MB_Group3_ dissemination capability [28, 33, 46]. Moreover, in ATG7-silenced cells, two migration-related pathways (proteoglycans and adherent junctions, respectively, see Supp. Fig. 4b, online resource) are found significantly downregulated. Prompted by this evidence, we decided to analyse the expression of epithelial-to-mesenchymal transition (EMT) markers, such as Snail (*SNAI1*) and Vimentin (*VIM*), which are usually highly expressed in MB_Group3_ cells [28]. Indeed, individual silencing of AMBRA1, BECLIN 1 or ATG7 (and after 40 μM CQ treatment) impairs the expression of the EMT markers analysed in this experiment (Fig. 5h). Moreover, we also detected a decreased migration and invasion capabilities of siAMBRA1-, siATG7- and treated D283-Med cells (Fig. 5i) (Supp. Fig. 5c, online resource), this indicating that both AMBRA1 and autophagy downregulation influence cell migration in MB. Last, since EMT is known to induce chemo-resistance in MB [7], we checked whether the combination of autophagy inhibition (mediated by CQ) and cisplatin (CIS, a commonly used chemotherapeutic agent in MB [71]) could affect MB-cell survival. Both cell death and viability were analysed after either individual CQ and CIS treatments, or their combination. As shown in Fig. 5j, k, the combinatory approach decreases D283-Med cell survival, this implying that such an approach might have future therapeutic implications. In summary, our data demonstrated that AMBRA1 manipulation or autophagy inhibition negatively impact on the major phenotypical hallmarks of MB_Group3_ cells.

### Downregulation of both AMBRA1 and autophagy inhibits MB_Group3_ aggressiveness in vivo

**Fig. 6:**
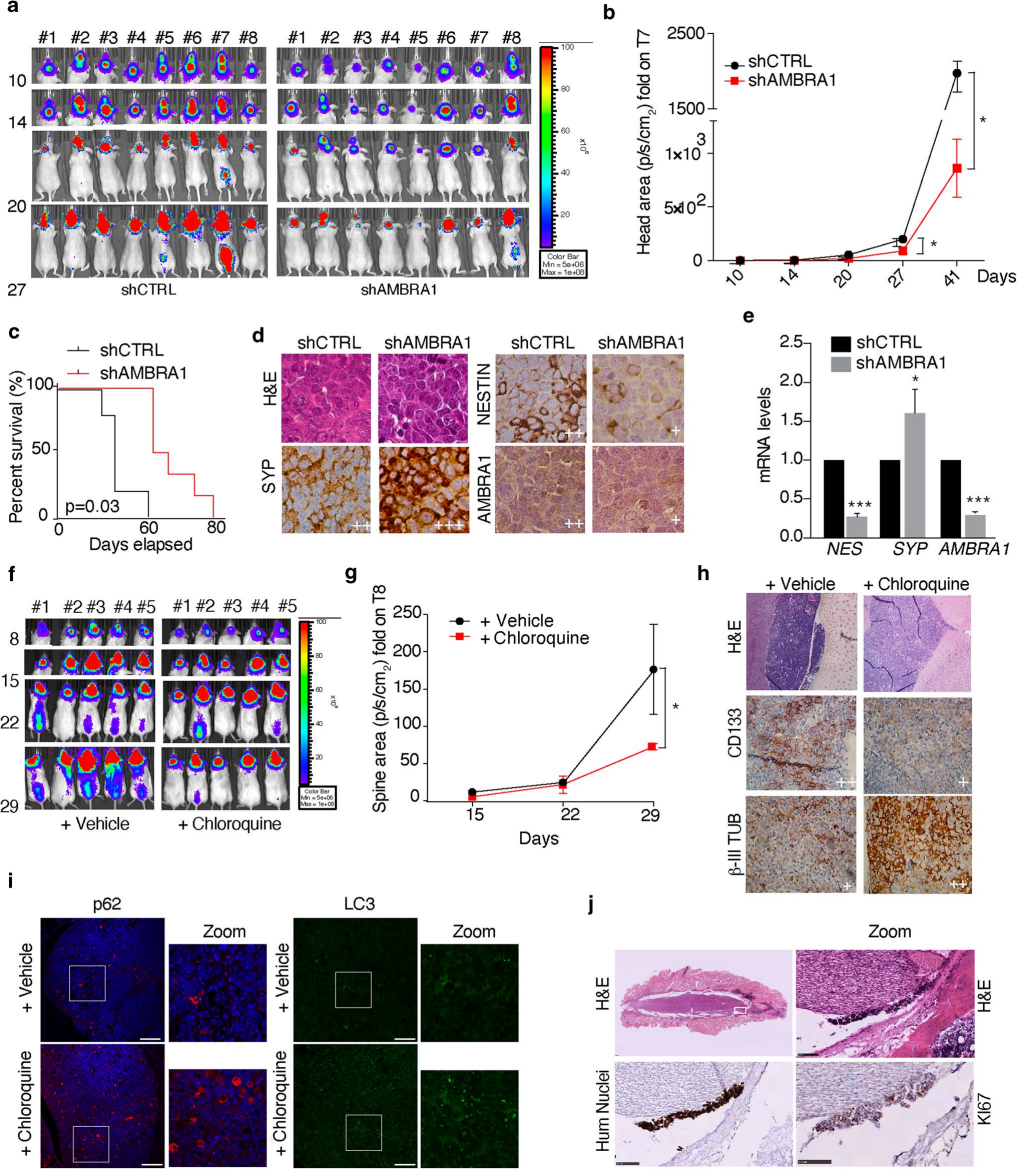
AMBRA1 downregulation and autophagy inhibition impact on MB_Group3_ aggressiveness in vivo. **a** Representative bioluminescent imaging of orthotopic xenograft experiment following injection into the fourth ventricle of nude mice (*n* = 16) of D283-Med-Luc cells. The mice were imaged at different time points for in vivo bioluminescence acquisition to monitor tumour growth. **b** Tumour growth according to quantified photon emission (ph/s) from the region of interest of mice represented in (**a)**. Data are the mean ± SEM (*n* = 8) and analysed by multiple *t*-test for each time points (*p < 0.05). **c** Kaplan–Meier analysis related to the experiment shown in (**a**). shCTRL *n* = 5; shAMBRA1 *n* = 6. *p* values were determined by the log-rank (Mantel-Cox) test. **d** Representative immunohistochemistry (60 ×) with indicated antibodies of paraffin-embedded cerebellar tumours generated by implanting D283-Med-Luc cells (shCTRL or shAMBRA1) into the fourth ventricle of nude mice, related to the experiment shown in (**a**). **e** qPCR analysis of *NESTIN*, *SYP* and *AMBRA1* were performed in cerebellar tumours generated by implanting D283-Med-Luc cells (shCTRL or shAMBRA1) into the fourth ventricle of nude mice D283-Med cells, related to the experiment shown in (**a**). Both *GADPH* and *B2M* were used as internal control. Data are expressed as the mean value ± SEM (*n* = 3). Data were analysed by unpaired Student's *t*-test (*p < 0.05, ***p < 0.001). **f** Representative orthotopic xenograft experiment following injection into the fourth ventricle of NOD scid gamma mouse mice (*n* = 10) of D283-Med-Luc cells. Mice were treated in vivo with CQ (60 mg/kg every day) intraperitoneally or with PBS as vehicle. The mice were imaged at different time points for in vivo bioluminescence acquisition, to monitor tumour growth. **g** Tumour growth according to quantified photon emission (ph/s) from the region of interest of mice represented in (**f**). Data are the mean ± SEM (*n* = 8) and analysed by one-way analysis of variance (ANOVA) followed by Tukey post hoc test (*p < 0.05). **h**, **i** Representative immunohistochemistry (40×) or immunofluorescence (20×-Zoom 60×, scale bar 100 μm) with indicated antibodies of paraffin-embedded cerebellar tumours generated by implanting D283-Med-Luc cells into the fourth ventricle of nude mice, related to the experiment shown in (**f**). **j** Representative immunohistochemistry (20×-Zoom 5×) with indicated antibodies of paraffin-embedded spinal cords related to the experiment shown in (**f**). Scale bar 100 μm

Due to the high levels of AMBRA1 expression in MB_Group3_, we decided to determine the effect of *AMBRA1* silencing on MB growth in vivo. The peritoneal metastatic D283-Med cell line represents an excellent model to study MB growth and metastatic dissemination in an orthotopic xenograft approach [85]. After engineering D283-Med cells *for* the constitutive expression of GFP and luciferase (shCTRL), we downregulated AMBRA1 by lentiviral infection (shAMBRA1) and orthotopically injected these cells into cerebella of athymic nude mice. We then followed tumour growth by weekly bioluminescent imaging (Fig. 6a) for 41 days. Notably, tumour masses arising from shAMBRA1 cells were significantly smaller than those deriving from control cells, as reported in Fig. 6b. Interestingly, when mice were analysed for a longer period, we found that shAMBRA1 cells-injected animals survive much longer (median survival: 62 days) than controls (*p* = 0.03; Fig. 6c). Both IHC and qPCR of primary tumour tissues from xenografted mice show inhibition of stem potential and proliferation (decreased NESTIN (NES) and Ki67 expression) and induction of cell differentiation (increased SYP expression) (Fig. 6d, e) (Supp. Figure 6a, online resource). Furthermore, staining for p62 and LC3 indicates that, in this context, autophagy is inhibited (Supp. Fig. 6a, online resource).

In light of these findings, we next wanted to investigate the effect of pharmacologic inhibition of autophagy by CQ on MB growth and migration in a second immune-compromised mouse model, NSG (NOD *scid* gamma mouse), which represents the most consistent model for studying cancer metastases [73]. First, we injected D283-Med-Luc cells into the cerebellum of NSG mice and, after confirmation of tumour engraftment by bioluminescent imaging (after 4 days), mice were randomized into two groups: those treated with either vehicle or CQ (60 mg/kg intraperitoneally every day). Animals were treated until they displayed tumour-associated morbidity and then euthanized (Fig. 6f). Although both groups generated similar sizes of primary cerebellar tumours (Supp. Fig. 6b, online resource), CQ-treated mice produced less detectable spinal metastases (Fig. 6g). Furthermore, IHC of the brain tumour tissues from these mice showed a decrease in CD133 and an increase in p62, LC3 *puncta* and β-III TUBULIN (β-III TUB) expression, respectively (Fig. 6h, i). Additionally, IHC of spinal cords dissected from the same mice displayed MB seeding cells that strongly express Ki67 (Fig. 6j). In sum, our data suggest that autophagy maintains stem cell potential and increases MB_Group3_ invasion ability, highlighting this pathway as a suitable and druggable target for MB.

### AMBRA1 depletion suppresses STAT3 signalling in MB_Group3_

Importantly, one key factor regulating both the maintenance and migration of CSCs is the signal transducer and activator of transcription 3 (STAT3), a factor involved in the development of multiple cancer types [8, 45]. RNA-seq of *AMBRA1* silencing in both D283-Med and CHLA-01-Med cells showed a shared downregulation pattern of c-MYC validated targets and STAT3 transcriptional activation (Supp. Fig. 7a, online resource). Given that (i) MB_Group3_ is characterized by upregulation of active STAT3 [32, 100]; (ii) STAT3 directly regulates markers of pluripotent stem cells (e.g., CD133, NANOG, SOX2, c-MYC) [31]; and, of the highest importance, (iii) AMBRA1 is a critical regulator of the activity of CULLINs [4, 5, 16], an E3-ubiquitin ligase family indirectly controlling STAT3 activity, we next hypothesized that STAT3 signalling was also involved in AMBRA1-dependent or autophagy-dependent MB_Group3_ stem potential.

**Fig. 7:**
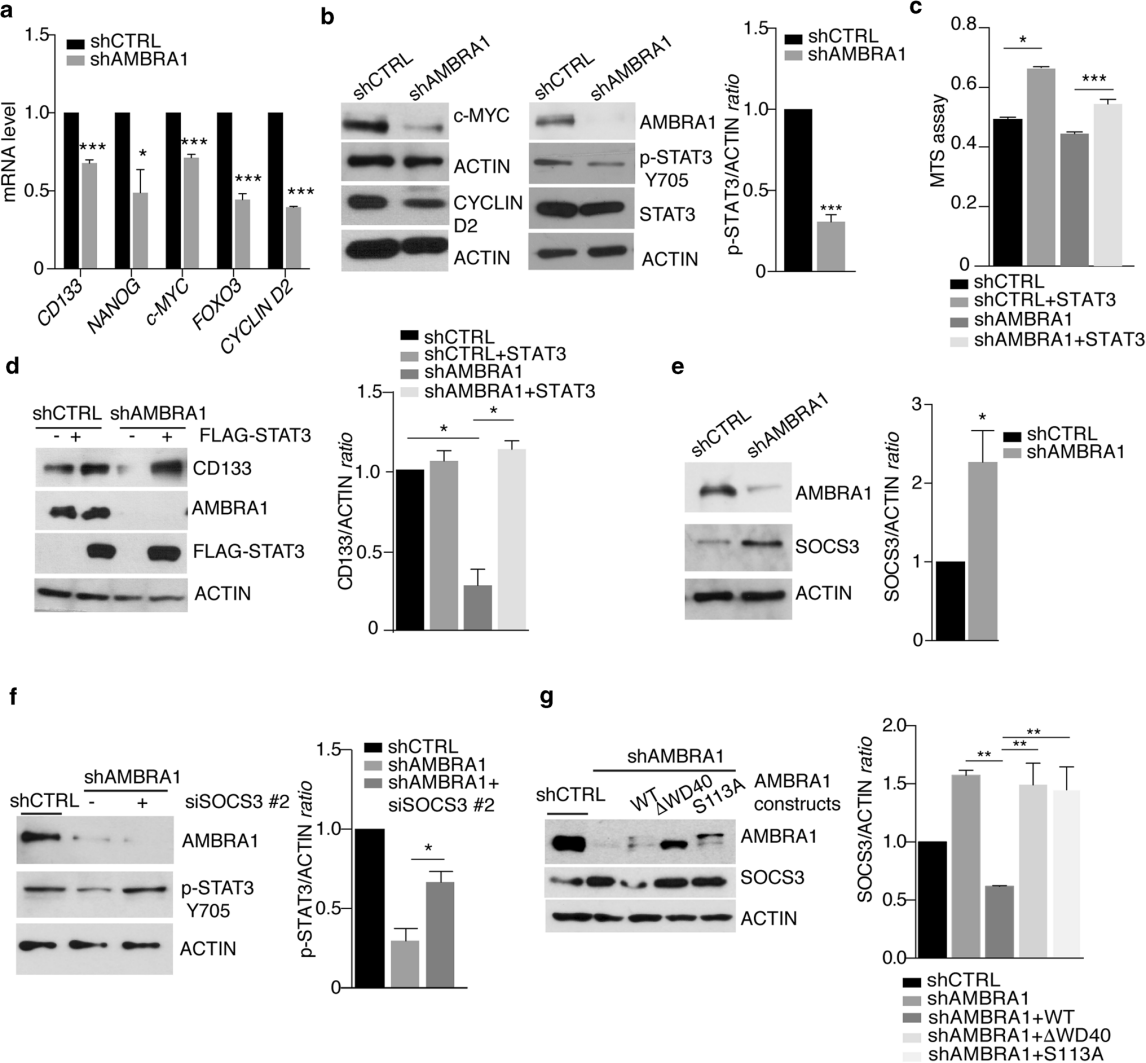
AMBRA1 depletion suppresses STAT3 signalling and c-MYC expression in MB_Group3_. **a** AMBRA1 expression was downregulated in D283-Med cells using specific RNAi oligonucleotides (siAMBRA1) or unrelated oligos as negative control (siCTRL). qPCR analysis of *CD133*, *NANOG*, c-*MYC*, *FOXO3* and *CYCLIN D2* was performed. Both *GADPH* and *B2M* were used as internal control. Data are expressed as the mean value ± SEM (*n* = 3). Data were analysed by unpaired Student's *t*-test (*p < 0.05, ***p < 0.001). **b** AMBRA1 expression was downregulated by lentiviral infection (shRNA AMBRA1) or negative control (shRNA CTRL). Levels of AMBRA1, c-MYC, CYCLIN D2, STAT3, p-STAT3 and ACTIN were analysed by WB. Densitometric analysis of p-STAT3 over ACTIN is also shown (right panel). Data are expressed as the mean ± SEM (*n* = 3) and analysed by unpaired Student's *t*-test (**p < 0.01, ***p < 0.001). **c** AMBRA1 expression was downregulated as in (**b**). Then, some of them were transfected with empty or STAT3-FLAG plasmids, respectively. Proliferation was assessed by MTS assay. Data are expressed as the mean ± SEM (*n* = 3) and data were analysed by one-way analysis of variance (ANOVA) followed by Tukey post hoc test. (*p < 0.05; ***p < 0.001). **d** D283-Med cells were treated as in (**c**). Levels of AMBRA1, CD133, STAT3 and ACTIN were analysed by WB. Densitometric analysis of CD133 over ACTIN is also shown (right panel). Data are expressed as the mean ± SEM (*n* = 3) and analysed by one-way analysis of variance (ANOVA) followed by Tukey post hoc test. (*p < 0.05). **e** D283-Med cells were treated as in (**b**). Levels of SOCS3 and ACTIN were analysed by WB. Densitometric analysis of SOCS3 levels over ACTIN is also shown. Data are expressed as the mean ± SEM (*n* = 4) and analysed by unpaired Student's *t*-test (*p < 0.05). **f** D283-Med cells were treated as in (**b**). In part of them, SOCS3 was downregulated using specific RNAi oligonucleotides (siSOCS3) or unrelated oligos. Levels of AMBRA1, p-STAT3 (Y705) and ACTIN were analysed by WB. Densitometric analysis of p-STAT3 levels over ACTIN is also shown. Data are expressed as the mean ± SEM (*n* = 3) and data were analysed by one-way analysis of variance (ANOVA) followed by Tukey post hoc test. **g** D283-Med cells were treated as in (**b**). In part of them, three different AMBRA1 plasmids (AMBRA1^WT^, AMBRA1^∆WD40^, AMBRA1^S113A^) were transfected. Levels of AMBRA1, SOCS3 and ACTIN were analysed by WB. Densitometric analysis of SOCS3 levels over ACTIN is also shown. Data are expressed as the mean ± SEM (*n* = 3) and data were analysed by one-way analysis of variance (ANOVA) followed by Tukey post hoc test

Indeed, in agreement with others [9, 32], we found that MB_Group3_ cells rely on STAT3, since genetic inhibition of *STAT3* by gene knockdown (after selection of the most efficient siRNAs) results into a significant decreased expression of stemness markers, such as c-*MYC* and *CD133* (Supp. Fig. 7b,c online resource), decreased proliferation (Supp. Fig. 7d, online resource), increased cell death (Supp. Fig. 7e, online resource) and decreased migration capabilities (Supp. Fig. 7f, online resource). Interestingly, we observed that *AMBRA1* downregulation in D283-Med cells decreases STAT3 transcriptional targets, such as *CD133*, *NANOG*, *FOXO3*, *CYCLIN D2* and, of the highest importance, c-*MYC* (Fig. 7a). Intriguingly, only a few of them (*CD133*, *NANOG*) decreased in *ATG7*-depleted cells (Supp. Fig. 7g, online resource). Moreover, in *AMBRA1*-deficient cells, we found a decrease of STAT3 activation by tyrosine-phosphorylation (Tyr 705) [21] and of the expression of STAT3 target genes in both D283-Med and CHLA-01-Med cells (Fig. 7b) (Supp. Fig. 7 h–k, online resource), including *c-MYC*, which does not occur at all after *ATG7* silencing (Supp. Fig. 7 g, k, online resource). To verify whether STAT3 was responsible for the decrease in both the expression of stemness markers and cell viability after *AMBRA1* downregulation, we overexpressed *STAT3* in *AMBRA1*-deficient cells. As shown in Fig. 7c, d and in Supp. Fig. 7l (online resource), STAT3 is able of significantly restoring its-target genes expression levels and cell viability after *AMBRA1* downregulation, this suggesting a crucial role for STAT3 in determining AMBRA1- function in MB context.

To unravel the mechanism behind this AMBRA1-mediated regulation, we set up to investigate the molecular axis that may lead to STAT3 activation. In more details, AMBRA1 has been shown to interact with the CRL4-DDB1 complex [that involves CULLIN 4 (CUL4)] to target Elongin C (ELOC), an essential adaptor protein that mediates the assembly of the CRL5-SOCS3 complex, which includes CUL5 [16]. This complex in turn influences the stability of Suppressor of Cytokines signalling 3 (SOCS3) [16, 34]. Indeed, SOCS3 inhibits STAT3 activation by acting on the JAK2 kinase-dependent phosphorylation on Tyr 705 [61]. We thus hypothesized that AMBRA1, besides regulating autophagy, could also control STAT3 activation by mediating the stability of its inhibitor SOCS3 in MB. Intriguingly, SOCS3 protein levels increase in *AMBRA1*-silenced cells, while a decrease was found at the mRNA level (Fig. 7e) (Supp. Fig. 7m, online resource). We then determined whether preventing SOCS3 accumulation in AMBRA1-deficient cells was sufficient to rescue STAT3 activation. After testing three different siRNAs against *SOCS3* (see Supp. Fig. 7n, online resource), we generated cells in which *AMBRA1* and *SOCS3* were co-depleted; as expected p-STAT3 is significantly decreased in cells upon *AMBRA1* downregulation, while siRNA-mediated silencing of *SOCS3* restores STAT3 phosphorylation, confirming the existence of an epistatic relationship among AMBRA1, SOCS3 and STAT3 (Fig. 7f). Finally, since AMBRA1 effect on CRL5 depends on the association between CRL4-DDB1 and AMBRA1 [16], we investigated whether the binding between AMBRA1 and DDB1 was crucial for SOCS3 regulation. To this aim, we used two specific AMBRA1 mutant constructs, deficient for its interaction with the CUL4-DDB1 complex (AMBRA1^∆WD40^ and AMBRA1^S113A^) [4]; as shown in Fig. 7g, after their overexpression in *AMBRA1*-silenced cells, an increased stability of SOCS3 was observed compared to wild-type form of AMBRA1. Altogether, we thus found that AMBRA1 controls STAT3 activity via SOCS3, independently of autophagy and via CRL4-DDB1, thereby increasing the levels of c-MYC and other stem cell markers.

### Combined targeting of autophagy and STAT3 signalling impairs MB_Group3_ progression

**Fig. 8:**
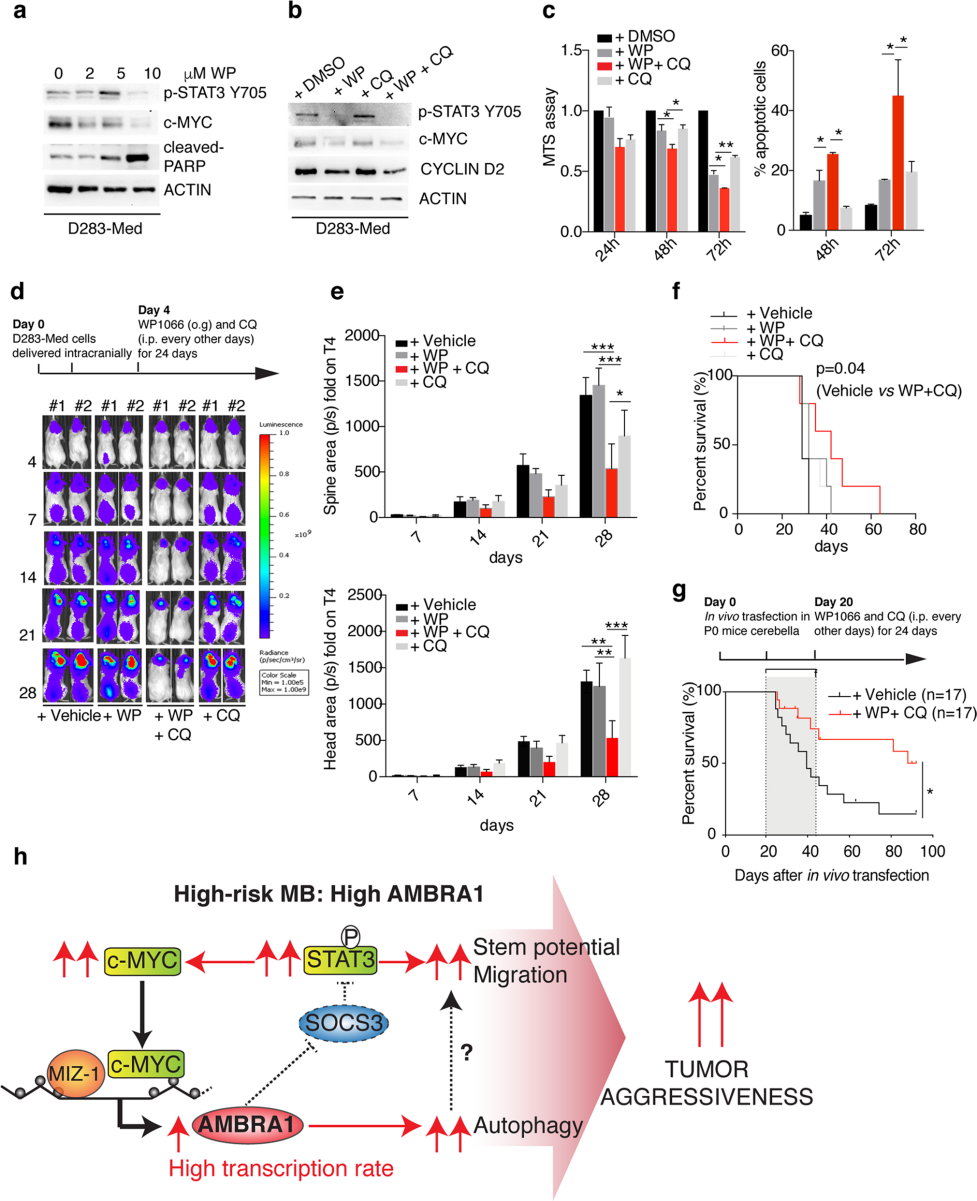
Combined targeting of autophagy and STAT3 signalling impairs MB_Group3_ progression. **a** D283-Med cells were treated with a range of WP1066 (WP) concentrations (2, 5, 10 µM). Levels of p-STAT3 (Y705), c-MYC, cleaved-PARP and ACTIN were analysed by WB. **b** D283-Med cells were treated with WP1066 (10 µM), CQ (40 µM) alone or in combination for 48 h. Levels of p-STAT3 (Y705), c-MYC, CYCLIN D2 and ACTIN were analysed by WB. **c** D283-Med cells were treated as in (**b**) for different time periods and proliferation was monitored by MTS assay (left). Data are expressed as the mean ± SEM (*n* = 6) and data were analysed by one-way analysis of variance (ANOVA) followed by Tukey post hoc test. (*p < 0.05; **p < 0.01; ***p < 0.001). Apoptosis was evaluated by flow-cytometry measuring Annexin V-Propidium iodure positive cells (right). Data are expressed as the mean ± SEM (*n* = 3) and data were analysed by one-way analysis of variance (ANOVA) followed by Tukey post-hoc test. (*p < 0.05; **p < 0.01). **d** Representative orthotopic xenograft experiment following injection into the fourth ventricle of NOD scid gamma mouse mice (*n* = 5) of D283-Med-Luc cells. After 4 days, mice were treated in vivo with vehicle, CQ (i.p. 60 mg/kg every other day), WP1066 (o.g. 40 mg/kg every other day) or combination of both. The mice were imaged at different time points for in vivo bioluminescence acquisition, to monitor tumour growth. **e** Tumour growth according to quantified photon emission (ph/s) from the region of interest of mice represented in (**d**). Data are the mean ± SEM (*n* = 5) and analysed by one-way analysis of variance (ANOVA) followed by Tukey post hoc test (*p < 0.05, **p < 0.01). **f** Kaplan–Meier survival curve related to the experiment shown in (**d**). *n* = 5. *p* value is determined by the log-rank (Mantel–Cox) test. *p = 0.04. **g** Kaplan–Meier survival curve of mice injected at P0 with c-Myc and Otx2-encoding vectors and then treated (P20) with vehicle or combination of both CQ (i.p. 60 mg/kg) and WP1066 (i.p. 30 mg/kg) every other day. *n* = 17. *p* value is determined by the log-rank (Mantel- Cox) test. *p = 0.0194. **h** In high-risk MB_,_ AMBRA1 is upregulated in a c-MYC/MIZ-1 dependent manner, thereby activating both the STAT3 pathway and autophagy, this leading to enhanced stem potential, migration and aggressiveness of MB

Due to the role of AMBRA1 in controlling both autophagy and STAT3 activity, and since STAT3 inhibition has been shown per se to impact autophagy [104], we speculated that inhibition of both STAT3 and autophagy could be more effective than either alone in MB treatment. To this aim, we used two STAT3 inhibitors, NSC 74859 (also known as S3I-201) that inhibits STAT3 dimerization [86] and WP1066, a STAT3 inhibitor that readily crosses the blood–brain-barrier [26, 38] that prevents STAT3 phosphorylation. First, a strong effect is observed upon both NSC 74,859 and WP1066 treatment, with decreased p-STAT3 levels and reduced expression of its target genes (c-*MYC* and *CYCLIN D2*) (Fig. 8a) (Supp. Fig. 8a, online resource). Interestingly, after pharmacological combination of both STAT3 and autophagy inhibition (by CQ 40 μM), we observed a significant decrease of proliferation and increased cell death when compared to STAT3 or autophagy inhibition alone (Fig. 8b, c) (Supp. Fig. 8b, c, online resource). Moreover, we observed an increased amount of LC3B-II in STAT3i-treated cells compared to vehicle-treated controls both basally and in the presence of CQ to inhibit flux (Supp. Fig. 8d, online resource), in line with the anti-autophagic functions of STAT3 [104].

To extend these in vitro findings to in vivo tumour growth, we evaluated the combination of STAT3 and CQ inhibition after injection of D283-Med-Luc cells into the cerebellum of NSG mice. In this experiment, both WP1066 and CQ were given every second day to reduce stress on the mice. As shown in Fig. 8d, e and Supp. Fig. 8e (online resource), brain tumors harvested from mice treated with the combination of WP1066 (40 mg/kg) and CQ (60 mg/kg) were significantly smaller than tumors from mice treated with STAT3 or autophagy inhibition alone at day 28. Additionally, the combination of both drugs induces less detectable spinal metastases. Also, mice treated with the combination of WP1066 and CQ exhibited a 13-day increased median survival compared with both vehicle-treated mice and CQ-treated mice, and a 10-day increased compared with WP treatment alone (Fig. 8f). We can thus conclude that concurrent inhibition of STAT3 and autophagy suppresses MB_Group3_ growth more efficiently than inhibition of STAT3 and CQ alone.

Since MB represents a very heterogeneous family of tumours requiring different therapeutic approaches to obtain reliable results, we decided to test the combination of both WP1066 and CQ in a MB_Group3_ mouse model, in which tumours are induced by c-Myc and orthodenticle homeobox 2 (Otx2) overexpression in P0 CD1 mice cerebella, as previously described [10, 57]. Mice treated with the combination of WP1066 (30 mg/kg) and CQ (60 mg/kg) for 24 days present a higher survival rate, when compared with vehicle-treated mice (Fig. 8g). Intriguingly, western blotting analysis of primary tumour tissues show a significant upregulation of Ambra1 protein levels, at variance with normal cerebellum (Supp. Fig. 8f, online resource), supporting the role of c-MYC overexpression in enhancing AMBRA1 levels.

In sum, we can conclude that upon c-MYC/MIZ-1-mediated upregulation of AMBRA1 transcription, this factor enhances both autophagy and STAT3-c-MYC signalling in high-risk MB. Molecular dissection of this axis primed us to demonstrate that acting on both pathways may represent a valid combined approach to counteract high-risk MB growth and spread (Fig. 8h).

## Discussion

As previously shown, AMBRA1 is an important factor at the crossroad between autophagy, development and cancer [18, 19, 50, 82]. Its intrinsically disordered structure (ID), indeed, allows AMBRA1 to have a great plasticity in protein–protein interactions and to mediate an extensive crosstalk between autophagy and other pathways [60]. In fact, besides autophagy, AMBRA1 acts as an essential co-factor for many E3 ligases belonging to both CULLIN and HECT E3-ubiquitin ligase families [4, 5, 16, 23]; however, the functional implications of this interplay in human diseases are still vastly unexplored. Of note, autophagy represents an emerging field in stem cell studies, being a putative controller of cell proliferation, cell differentiation and migration and, as for autophagy importance, cancer stem cells are not an exception [17, 62]. Interestingly, despite numerous efforts to counteract MBSCs aggressiveness, the molecular pathways and individual players involved remain largely unknown. In this study, we show that AMBRA1 is strongly upregulated in MB_Group3_ stem cells and that its silencing has a strong effect on stem potential, viability and migration. This phenotype is recapitulated only in part by inhibition of autophagy, through a mechanism that remains to be elucidated, this suggesting the involvement of multiple specific regulations mediated by AMBRA1. Indeed, upon AMBRA1 depletion, a dramatic inhibition of STAT3 signalling occurs, through the stabilization of SOCS3, with this confirming that MB stem potential and cell survival also depends in part on AMBRA1 regulation.

By dissecting the molecular details of AMBRA1 transcriptional enhancement, fundamental for MB_Group3_ cell stem potential and growth, our work identifies a new *AMBRA1*-c-*MYC* regulatory axis. On one hand, we propose that c-MYC overexpression induces *AMBRA1* transcription in a MIZ-1-dependent manner.

It is important to consider that c-MYC-MIZ-1 binding is critical for the development of MB_Group3_, modulating the expression of specific genes that maintain the identity of these tumours and distinguish them from other MB subgroups [95]. Interestingly, c-MYC and MIZ-1 are found at the promoters of 1,736 downregulated and 1,822 upregulated genes in MB_Group3_ relative to MB_SHH_. On the other hand, loss of AMBRA1 in MB_Group3_ is sufficient to decrease the expression of STAT3-dependent *c-MYC* transcription and consequently to impair cell viability. Altogether, these findings open a novel scenario for targeting c-MYC transcription, and it would be of the highest importance to check whether such an AMBRA1 dependence could be detected in other different c-MYC-driven tumours, such as leukaemia or gliomas [43, 80]. Intriguingly, one of STAT3 target genes is the transcriptional factor FOXO3 [68]. Loss of FOXO3 is known to compromise the pool of neural stem cells, decrease their self-renewal and impair their ability to differentiate into neural specific lineages [75]. Interestingly, FOXOs have been reported to regulate gene expression of a large number of genes encoding proteins involved in autophagy [81, 96].

Moreover, considerable evidence supports the crucial roles of STAT3 in human diseases, particularly in immune and inflammatory disorders, infections and cancer [12]. Increasing emphasis is being placed on developing direct STAT3 inhibitors for clinical application and some clinical trials in advanced cancer are ongoing [52, 94]. In pre-clinical studies on MB, inhibition of the persistent STAT3 signalling pathway blocks proliferation and migration and induces apoptosis, even though little is known about putative upstream regulators of this signalling [14, 100]. Notably, the novel orally administered STAT3 inhibitor (WP1066) [26, 38] has been developed with excellent blood–brain-barrier penetration and a Phase I Trial (NCT01904123) is currently ongoing against recurrent and malignant glioma.

Patients with high-risk MB are particularly resistant to the treatments that currently exist. A major barrier is temporal heterogeneity and patients with MB_Group3_ or MB_Group4_ die almost exclusively as a result of metastatic disease, which suggests that a treatment-resistant clone in the metastatic compartment results in relapse [74]. Very recently, a study identified IL-6 as a promoter of STAT3 signalling in development of drug resistance in MB_Group3_ [91]_._ Improved knowledge of some pathways (drug export, DNA repair and apoptotic inhibition) in MB has provided novel emerging therapeutic targets for circumventing chemoresistance and for restoring chemotherapeutic efficacy. For example, PARP inhibitors have shown considerable promise in the treatment of MB and inhibition of PARP has been shown to enhance TMZ activity in vitro and in vivo [20]. Additionally, MB CSCs are also known to contribute to treatment failure through multiple drug resistance mechanisms (i.e., the activation of pro-survival/anti-apoptosis pathways) [40]. CSCs are known to be significantly involved in autophagy-mediated chemo-resistance, combining their already aggressive phenotype to autophagy activation, which further enhances tumour drug-resistance [62]. Experimental observations and clinical trials suggest that autophagy inhibition may be used for anti-CSCs therapy even if, in most cancers, the benefits of CQ monotherapy is very limited. Our experimental observations suggest that pharmacological inhibition of autophagy and STAT3 may be used in combinatorial approaches for treating stemness-dependent MB aggressiveness. It remains to be investigated whether combining agents able to inhibit autophagy with radiotherapy or other chemotherapeutic drugs would result in even greater effects on MB growth.

On the same line, given the growing importance of patient stratification in MB towards a more personalized treatment [27], and considering our data on the correlation existing between MB_Group3_ overall survival and AMBRA1 expression, measurement of AMBRA1 levels could represent a novel relevant prognostic tool. Interestingly, Cavalli et al*.* have recently subcategorized 144 MB_Group3_ into G3α (46.5%), G3β (25.7%) and G3γ (27.8%) subgroups with distinct genetic aberrations, activated pathways and clinical outcomes [13]. G3α mostly affects infants and young children up to 10 years of age and, clinically, G3α and G3β exhibit a more favorable prognosis when compared with G3γ; also, G3α and G3γ have a similar frequency of metastatic dissemination at diagnosis. When analysing these MB subtypes, we identified a key role for AMBRA1 expression in G3α, in which high AMBRA1 levels correlate with high CD133 levels and with poor patient survival. Intriguingly, by analysing AMBRA1 correlation with patient survival in both MB_Group3_ and G3α, we found that high AMBRA1 is significantly associated with poor prognosis by univariate and multivariate analyses independently from c-MYC levels. Our findings indicate that AMBRA1 is an independent factor for poor prognosis in these subtypes, and it may be helpful in predicting the disease outcome and for managing MB patients. Until now, subgroup-targeted therapies are yet to be found; however, treatment choices based on specific subgroup features have certainly the potential to impact patient survival and avoid long-term side effects.

## Supplementary Information

Below is the link to the electronic supplementary material.

Supplementary file1 (PDF 5206 KB)
