# Supplementary material for: Targeting cancer stem cells in medulloblastoma by inhibiting AMBRA1 dual function in autophagy and STAT3 signalling
Source: Acta Neuropathol. 2021 Jul 24;142(3):537–64. doi: 10.1007/s00401-021-02347-7 (PMC8357694; doi:10.1007/s00401-021-02347-7)
Supplement: Supplementary file 1 — Supplementary file1 (PDF 5206 KB) [file 401_2021_2347_MOESM1_ESM.pdf]

Supp. Figure 1

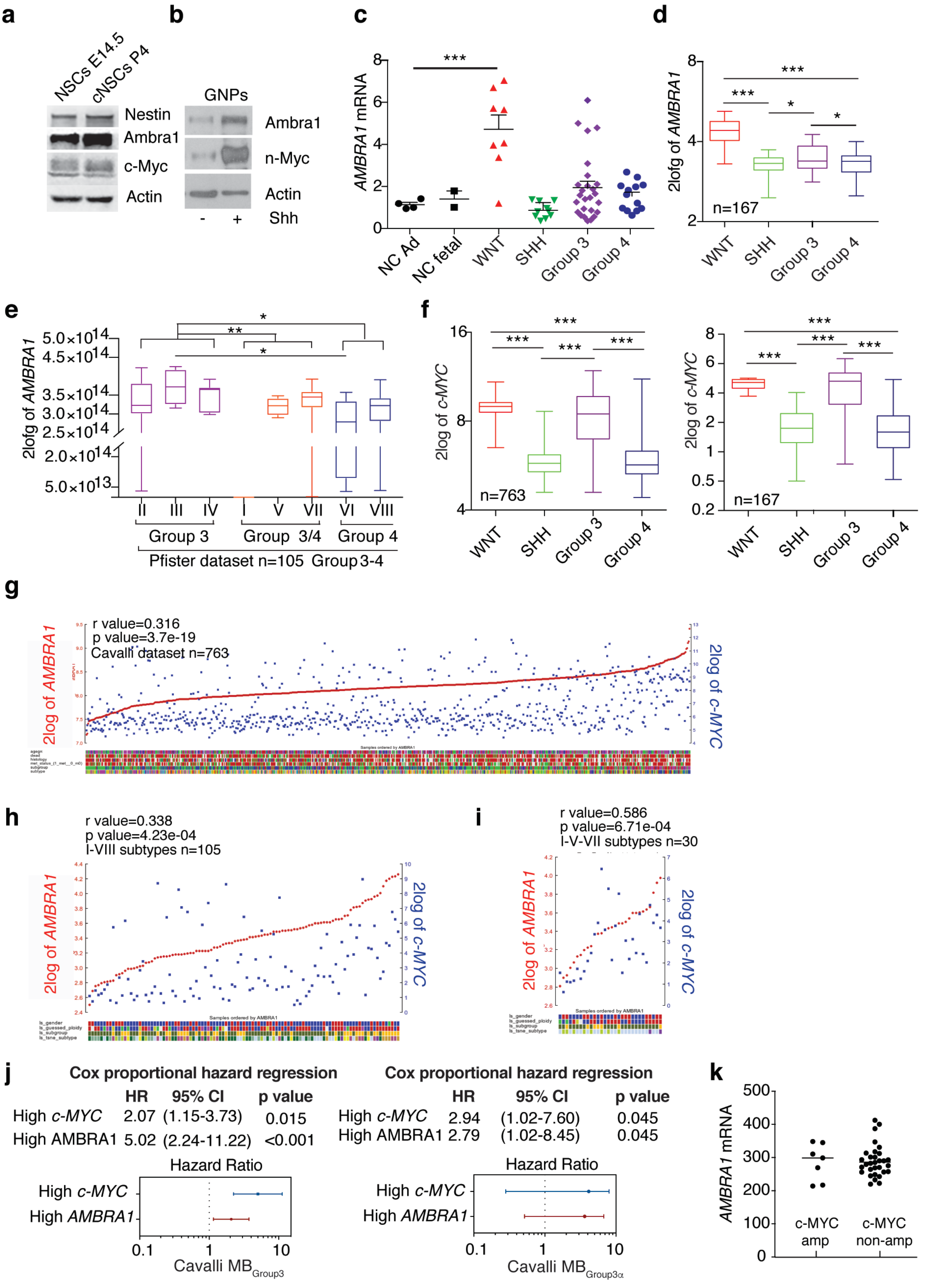

Supp. Figure 2

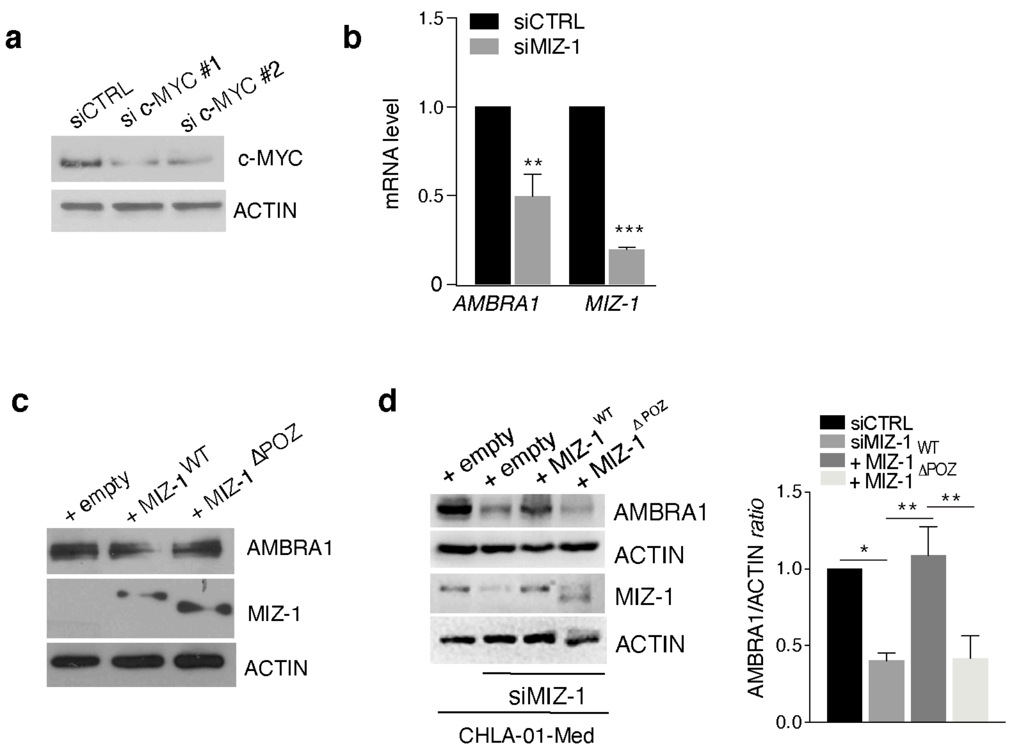

Supp. Figure 3

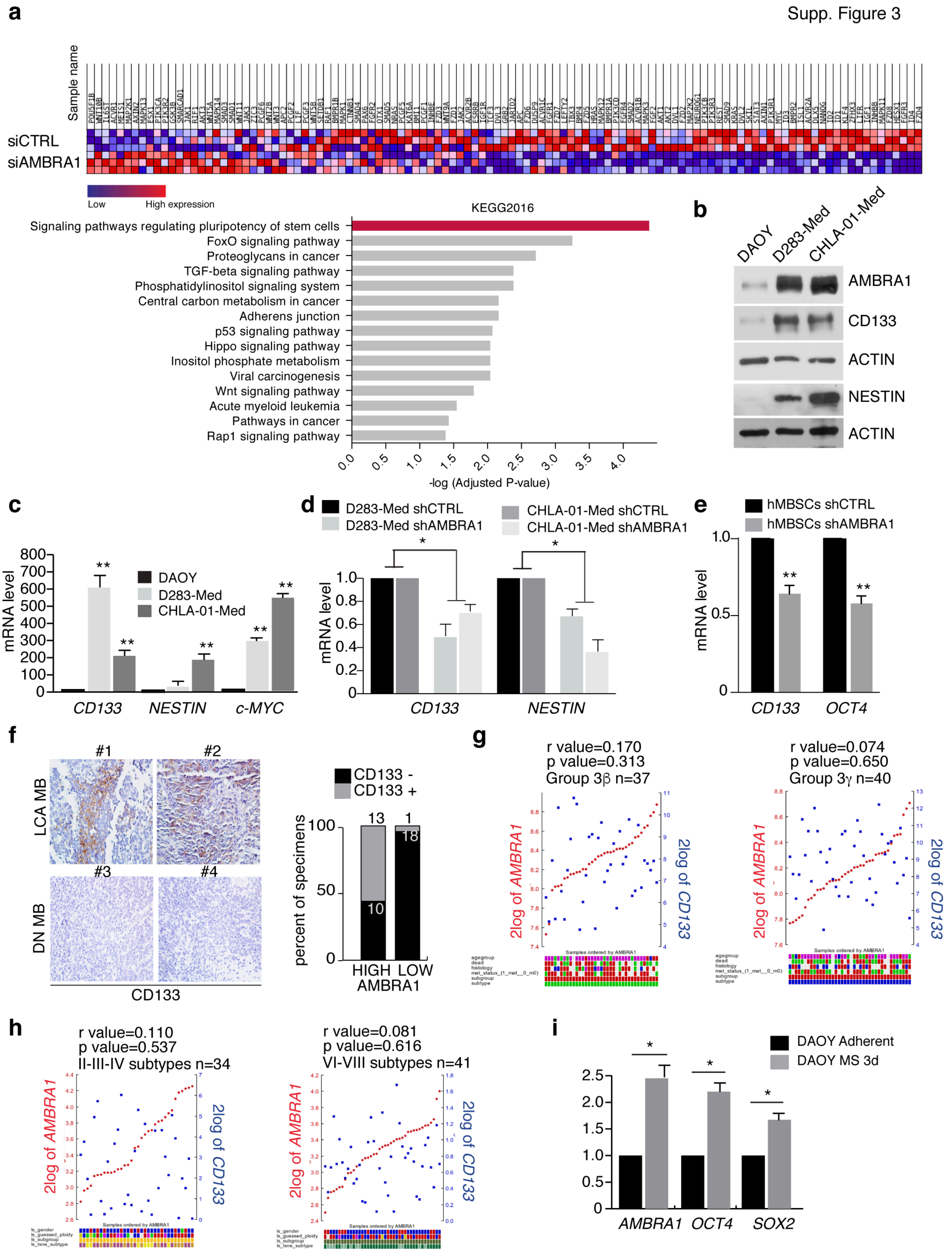

Supp. Figure 3

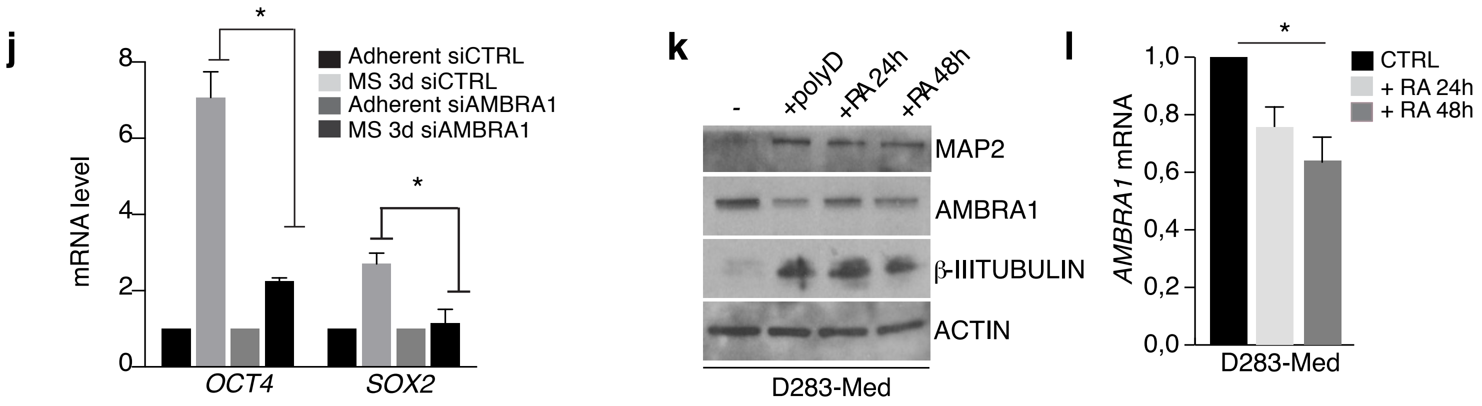

Supp. Figure 4

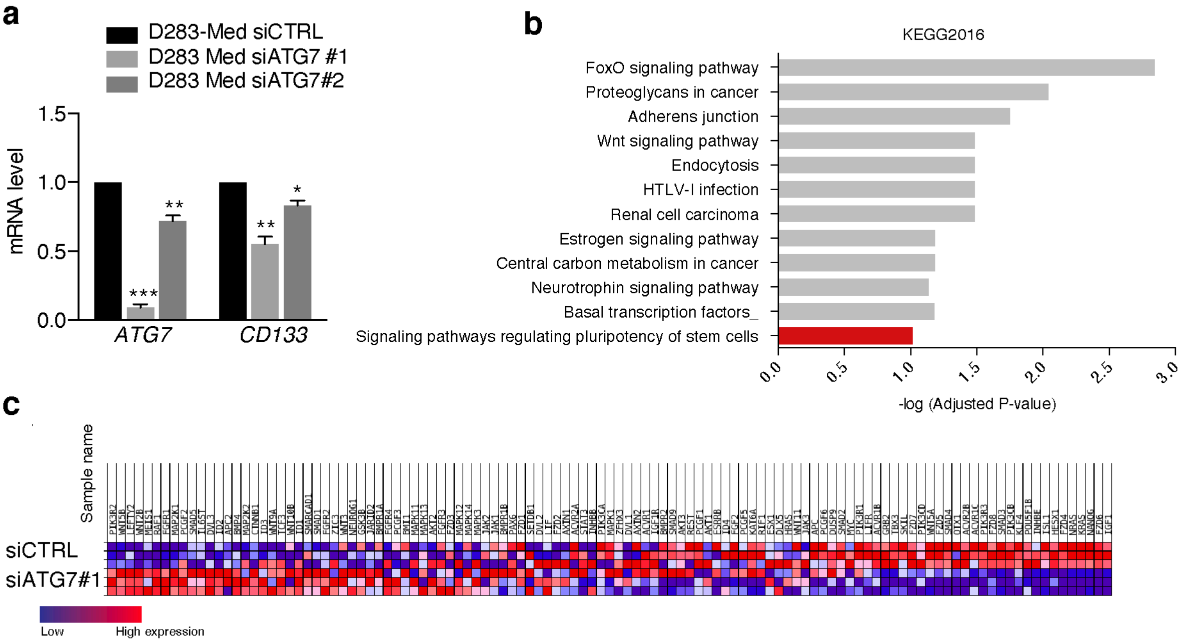

Supp. Figure 5

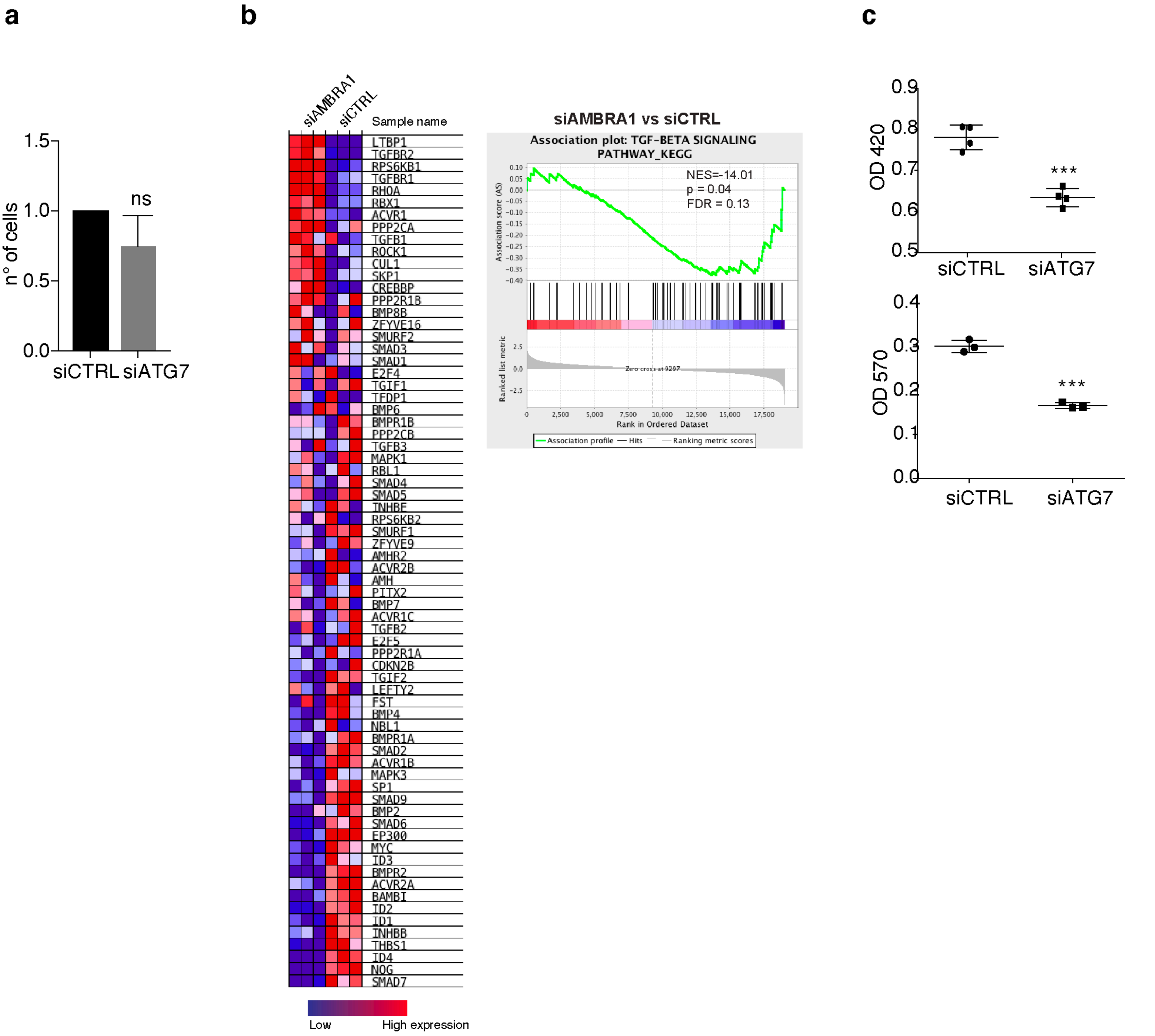

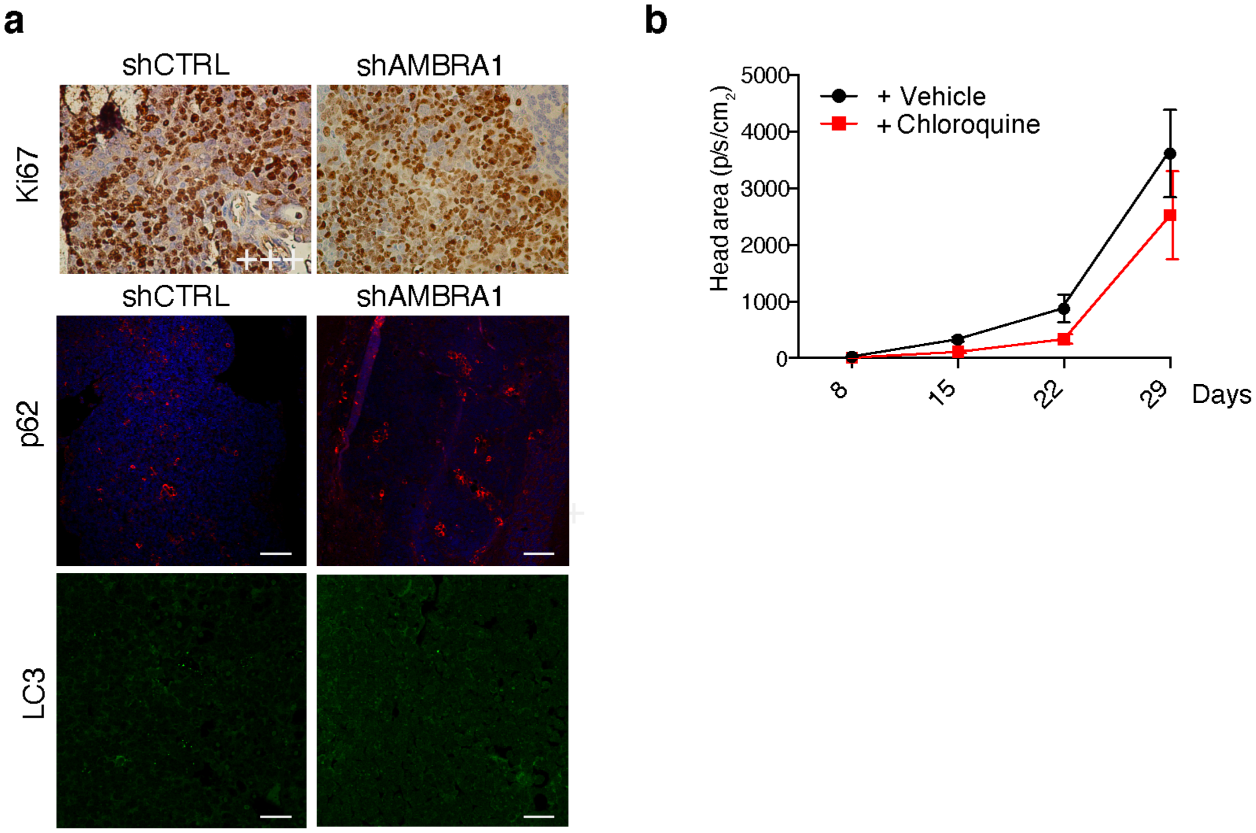
a
shCTRL
shAMBRA1
Ki67
shCTRL
shAMBRA1
p62
LC3
b
Head area (p/s/cm2)
+ Vehicle
+ Chloroquine
5000
4000
3000
2000
1000
0
8
15
22
29
Days

Supp. Figure 7

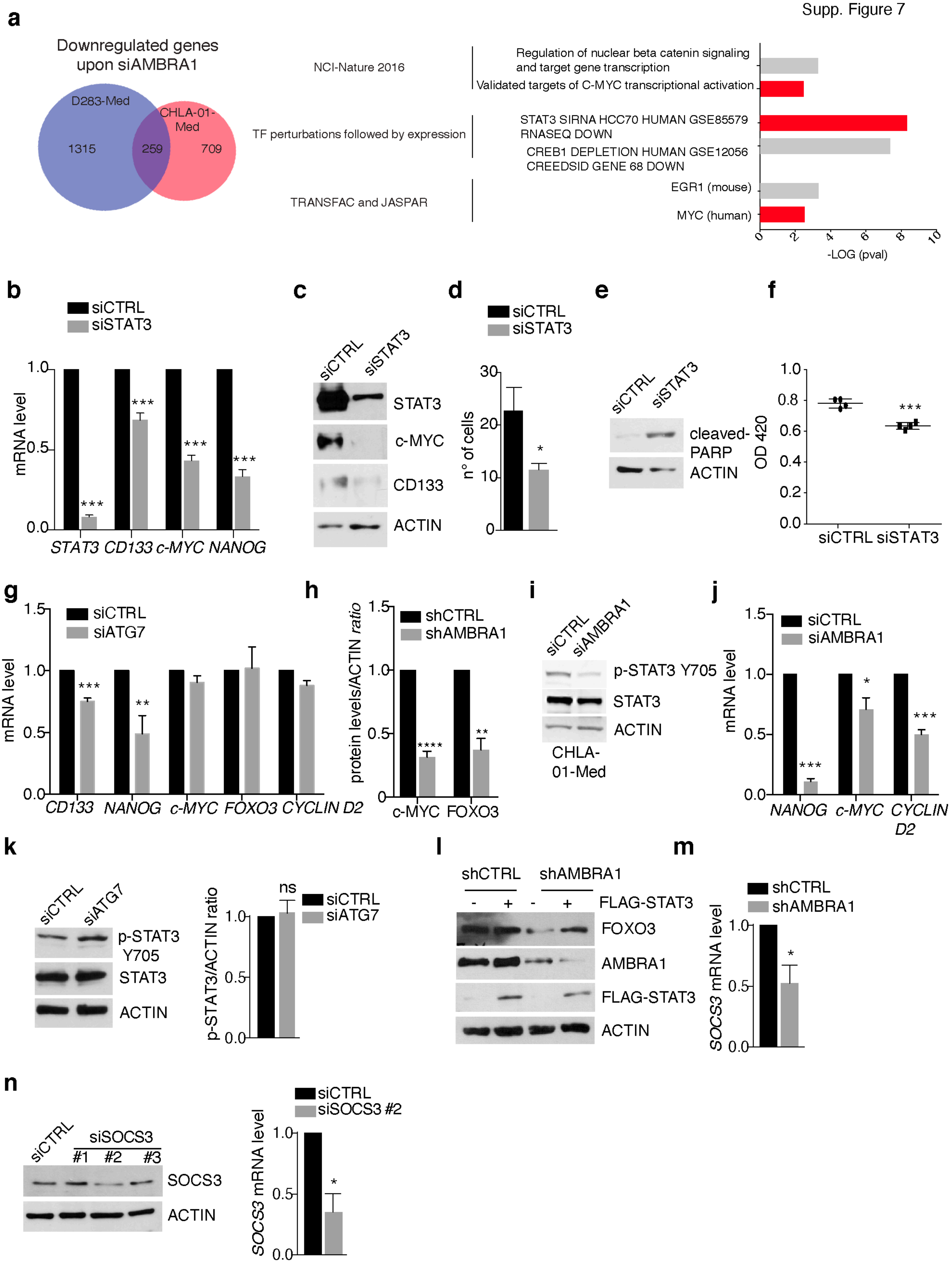

Supp. Figure 8

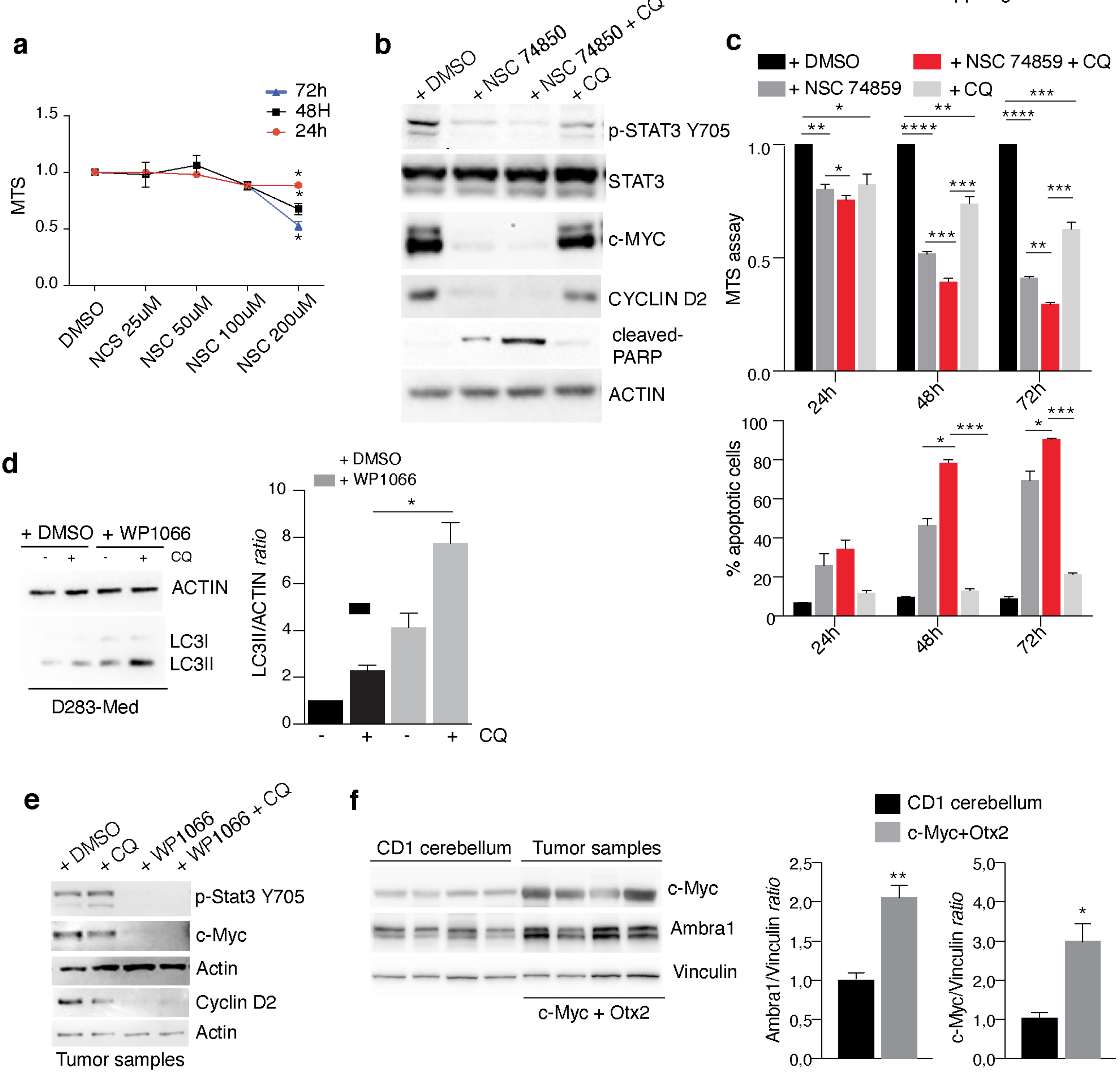

Supplementary Figure legends

Supp. Figure 1: AMBRA1 and c-MYC expression in MB tumour samples and cell lines

a) Neural stem cells (NSCs) were isolated from Medial Ganglionic Eminences at E14.5 while cerebellar NSCs (cNSCs) were obtained from postnatal 4-day-old wild-type mice (P4). Levels of Nestin, Ambra1, c-Myc and Actin were analysed by WB.

b) Cerebellar granule neuron precursors (GNPs) (prepared from 4-day-old mice) were isolated and treated with Shh (3 µg/ml) for up to 72 h. Levels of Ambra1, n-Myc and Actin were analysed by WB.

c) qPCR analysis of *AMBRA1* mRNA among adult and fetal cerebellum and in MB patients. Data are expressed as the mean value ± SEM. Data were analysed by one-way analysis of variance (ANOVA) followed by Tukey post-hoc test (***p<0.001).

d) RNA log2 expression of *AMBRA1* derived from the publicly available dataset Pfister (167 samples, fpkm normalized, mb500rs1 chip), grouped according to the molecular subgroup disease variants. Data were analysed by one-way analysis of variance (ANOVA) followed by Tukey post-hoc test (*p<0.05, ***p<0.001).

e) RNA log2 expression of *AMBRA1* derived from the publicly available dataset Pfister (105 samples, fpkm normalized, mb500rs1 chip), grouped according to the 8 molecular subtypes disease variants. Data were analysed by non-parametric ANOVA test (Kruskal-Wallis test) (*p<0.05, **p<0.01).

f) RNA log2 expression of c-*MYC* derived from two publicly available datasets: Cavalli (763 samples, fpkm normalized, mb500rs1 chip) and Pfister (167 samples, fpkm normalized, mb500rs1 chip), grouped according to the molecular subgroup disease variants. Data were analysed by one-way analysis of variance (ANOVA) followed by Tukey post-hoc test (***p<0.001).

g) Gene expression correlation analyses between transcripts for *AMBRA1* and *c-MYC* in MB samples from the Cavalli dataset (n=763 tumour samples). Statistically significant positive correlation is shown between these genes (r=0.316, p=3.7e-19).

h) Gene expression correlation analyses between transcripts for *AMBRA1* and *c-MYC* in MB samples from the Pfister dataset (I-VIII subtypes n=105). Statistically significant positive correlation is shown between these genes (r=0.338 p=4.23e-04).

i) Gene expression correlation analyses between transcripts for *AMBRA1* and *c-MYC* in MB samples from the Pfister dataset (I-V-VII subtypes n=30 respectively). Statistically significant positive correlation is shown between these genes (r=0.586 p=6.71e-04).

j) Cox proportional hazard regression was used to determine the independent effect of high-level of *AMBRA1* and *c-MYC* expression on overall survival (OS) in MB_{Group3} and in G3α respectively from Cavalli dataset.

k) *AMBRA1* expression in G3γ subtype (derived from Cavalli dataset), divided according to the *c-MYC* amplification status.

Supp. Figure 2: Role of c-MYC/MIZ-1 complex in regulating AMBRA1 expression *in vitro*

a) c-MYC expression was downregulated in D283-Med cells using two different RNAi oligonucleotides (sic-MYC#1,#2) or unrelated oligos as negative control (siCTRL). Levels of c-MYC and ACTIN were analysed by WB.

b) MIZ-1 expression was downregulated in D283-Med cells using specific RNAi oligonucleotides (siMIZ-1). Both *AMBRA1* and *MIZ-1* mRNA levels were analysed by qPCR. *GADPH* and *B2M* were used as internal control. Data are expressed as the mean value ± SEM (n= 3). siCTRL was arbitrarily defined as 1.00. Data were analysed by unpaired Student's t-test (**p<0.01, ***p<0.001).

c) D283-cells were transfected with empty, MIZ-1^{WT} or MIZ-1$^{\Delta POZ}$ plasmids respectively. Levels of AMBRA1, MIZ-1 and ACTIN were analysed by WB.

d) MIZ-1 expression was downregulated in CHLA-01-Med cells using specific RNAi oligonucleotides (siMIZ-1). Then, some of them were transfected with empty, MIZ-1^{WT} or MIZ-1$^{\Delta POZ}$ plasmids respectively. Levels of AMBRA1, MIZ-1 and ACTIN were analysed by WB. Densitometric analysis of AMBRA1 levels over ACTIN is also shown. Data are expressed as the mean ± SEM (n= 4) and analysed by one-way analysis of variance (ANOVA) followed by Tukey post-hoc test (*p<0.05; ****p<0.01).

Supp. Figure 3: Analyses of AMBRA1-dependent role in MB stem potential

a) A heatmap showing the expression of representative pluripotency-related genes in D283-Med cells with or without AMBRA1 knockdown (siAMBRA1). Data are generated from RNA-seq analysis. Below, KEGG enrichment analysis of differentially expressed pathways in AMBRA1-depleted cells. Data are generated from RNA-seq.

b) AMBRA1, CD133, NESTIN and ACTIN protein levels were analysed by WB respectively in different MB cell lines.

c) qPCR of *CD133*, *NESTIN* and c-*MYC* in different MB cell lines. Both *GADPH* and *B2M* are used as internal control. Data are expressed as the mean value ± SEM (n= 3). Data

were analysed by one-way analysis of variance (ANOVA) followed by Tukey post-hoc test. (**$p<0.01$).

d) qPCR analyses of *CD133* and *NESTIN* in both D283-Med and CHLA-01-Med depleted for AMBRA1 (siAMBRA1). Data are expressed as the mean value ± SEM (n= 3). Data were analysed unpaired Student's t-test

e) qPCR analyses of *CD133* and *OCT4* in primary human MBSCs after AMBRA1 lentiviral downregulation (shAMBRA1). Data are expressed as the mean value ± SEM (n= 3). Data were analysed unpaired Student's t-test.

f) Representative IHC images in low magnification fields (40X) showed CD133 expression in two human desmoplastic/nodular (DN) and large cell/anaplastic (LCA) MBs respectively. Graphical display of IHC data for high or low AMBRA1 expression relative to level of CD133 is also shown (right).

g-h) Gene expression correlation analyses between transcripts for *AMBRA1* and *CD133* in MB samples from both the Cavalli (G3β and G3γ) and the Pfister (II+III+IV and VI+VIII subtypes) datasets respectively.

i) DAOY cells were cultured as MS for 3 days, *AMBRA1*, *OCT4* and *SOX2* mRNA levels were analysed by Real-time PCR. GADPH and B2M were used as internal control. Data are expressed as the mean ± SEM (n= 3) and analysed by unpaired Student's t-test (*$p<0.05$).

j) AMBRA1 expression was downregulated in DAOY cells using specific RNAi oligonucleotides (siAMBRA1) or unrelated oligos as negative control (siCTRL). After 24h, cells were cultured as medullospheres. Both *SOX2* and *OCT4* mRNA levels were analysed by qRT-PCR. *GADPH* and *B2M* were used as internal control. Data are expressed as the mean value ± SEM (n= 3). Data were analysed by one-way analysis of variance (ANOVA) followed by Tukey post-hoc test. (*$p<0.05$).

k) D283-Med cells were treated by retinoic acid (RA) for two different time points. Levels of AMBRA1, MAP2, β-III TUBULIN and ACTIN were analysed by WB.

l) D283-Med cells were treated as in (**i**). *AMBRA1* expression was analysed. *GADPH* and *B2M* were used as internal control. Data are expressed as the mean ± SEM (n= 3) and analysed by unpaired Student's t-test (*$p<0.05$).

Supp. Figure 4: Correlation between autophagy and stem potential

a) qPCR analyses of *CD133* and *ATG7* in D283-Med cells after ATG7 downregulation with two different specific siRNAs (ATG7#1,#2). Both *GADPH* and *B2M* were used as internal

control. Data are expressed as the mean value ± SEM (n= 3). Data were analysed by unpaired Student's t-test (*p<0.05, **p<0.01).

b) KEGG enrichment analysis of differentially expressed pathways in ATG7-depleted cells. Data are generated from RNA-seq.

c) A heatmap showing the expression of representative pluripotency-related genes in D283-Med cells with or without ATG7 knockdown. Data are generated from RNA-seq analysis.

Supp. Figure 5: Role of both AMBRA1 and autophagy in MB proliferation and migration

a) Proliferation was assessed by MTS assay in ATG7-depleted D283-Med cells. Data are expressed as the mean ± SEM (n= 3) and analysed by unpaired Student's t-test. ns= no significant.

b) A heatmap showing the expression of representative TGF-β signalling genes in D283-Med cells with or without AMBRA1 knockdown. Data are generated from RNA-seq analysis. Right, GSAA enrichment plot showing that loss of AMBRA1 in D283-Med cells (3 independent experiments) results in downregulation of TGF-β pathway.

c) D283-Med cells were treated as in (**a**) and then plated for both migration and invasion assay. Data are expressed as the mean value ± SEM (n= 4). Data were analysed by unpaired Student's t-test (***p<0.001).

Supp. Figure 6: Role of both AMBRA1 and autophagy in MB aggressiveness *in vivo*

a) Representative immunohistochemistry (40X) and immunofluorescence (20X) respectively with indicated antibodies of paraffin-embedded cerebellar tumours generated by implanting D283-Med-Luc cells (shCTRL or shAMBRA1) into the fourth ventricle of nude mice.

b) Tumour growth according to quantified photon emission (ph/s) from the region of interest of mice analysed in Figure 6**f**. Data are the mean ± SEM (n= 5) and analysed by one-way analysis of variance (ANOVA) followed by Tukey post-hoc test.

Supp. Figure 7: Analysis of the crosstalk among STAT3, AMBRA1 and autophagy in MB_{Group3} cells

a) Left. Venn diagram for downregulated expressed genes (padj<0.05) in both D283-Med and CHLA-01-Med cells, after AMBRA1 downregulation by specific RNAi oligonucleotides (siAMBRA1). Right. We used the gene-set enrichment tool Enrichr to analyse common downregulated genes between the two cells lines, interrogating “TRANSFAC and JASPAR”,

"Transcriptional factor perturbations Followed by Expression" and "NCI-Nature 2016" database. Per each group, only the two top results are shown.

b) *CD133*, *NANOG*, c-*MYC* and *STAT3* mRNA levels were analysed in D283-Med cells after STAT3 downregulation by specific RNAi oligonucleotides (siSTAT3) or unrelated oligos as negative control (siCTRL). Data are expressed as the mean value ± SEM (n= 3). Data were analysed by unpaired Student's t-test (*p<0.05, ***p<0.001).

c) D283-Med cells were treated as in (**b**). Levels of STAT3, c-MYC, CD133 and ACTIN were analysed by WB.

d) D283-Med cells were treated as in (**b**). After 48h, cells were counted. Data are expressed as the mean value ± SEM (n= 3). Data were analysed by unpaired Student's t-test (*p<0.05).

e) D283-Med cells were treated as in **(b**). Levels of cleaved-PARP and ACTIN were analysed by WB.

f) D283-Med cells were treated as in (**b**) and then plated for migration assay. Data are expressed as the mean value ± SEM (n= 4). Data were analysed by unpaired Student's t-test (***p<0.001).

g) qPCR analyses of *CD133*, *NANOG*, c-*MYC*, *FOXO3*, and CYCLIN D2 in D283-Med cells after ATG7 downregulation (siATG7). Both *GADPH* and *B2M* were used as internal control. Data are expressed as the mean value ± SEM (n= 3). Data were analysed by unpaired Student's t-test (*p<0.05, ***p<0.001).

h) Densitometric analyses (related to Fig. 7**b**) of both c-MYC and FOXO3 over ACTIN are shown (right panel). Data are expressed as the mean ± SEM (n= 3) and analysed by unpaired Student's t-test (**p<0.01, ***p<0.001)

i) AMBRA1 was downregulated by specific RNAi oligonucleotides (siAMBRA1) or unrelated oligos as negative control (siCTRL) in CHLA-01-Med cells. Levels of STAT3, p-STAT3 and ACTIN were analysed by WB.

j) *NANOG*, c-*MYC* and *CYCLIN D2* mRNA levels were analysed in CHLA-01-Med cells after AMBRA1 downregulation by specific RNAi oligonucleotides (siSTAT3) or unrelated oligos as negative control (siCTRL). Data are expressed as the mean value ± SEM (n= 3). Data were analysed by unpaired Student's t-test (*p<0.05, ***p<0.001).

k) D283-Med cells were treated as in (**g**). Levels of STAT3, p-STAT3 and ACTIN were analysed by WB. Densitometric analysis of p-STAT3 levels over ACTIN is also shown (right panel).

l) AMBRA1 expression was downregulated by lentiviral infection (shRNA AMBRA1) or negative control (shRNA CTRL). Then, some of them were transfected with empty or

STAT3-FLAG plasmids respectively. Levels of AMBRA1, FOXO3, STAT3 and ACTIN were analysed by WB.

m) *SOCS3* expression was analysed in AMBRA1-depleted D283-Med cells. Both *GADPH* and *B2M* were used as internal control. Data are expressed as the mean value ± SEM (n= 3). Data were analysed by unpaired Student's t-test (*p<0.05).

n) SOCS3 expression was downregulated in D283-Med cells using three different RNAi oligonucleotides (sic-SOCS3#1,#2, #3) or unrelated oligos as negative control (siCTRL). Levels of SOCS3 and ACTIN were analysed by WB. *SOCS3* expression was analysed in SOCS3#2-depleted D283-Med cells. *GADPH* was used as internal control. Data are expressed as the mean value ± SEM (n= 3). Data were analysed by unpaired Student's t-test (*p<0.05).

Supp. Figure 8: Both STAT3 and autophagy inhibition affects MB cells survival and autophagy

a) D283-Med cells were treated with a range of NSC 74859 concentrations (NSC, 25 200 μM) and proliferation was monitored by MTS assay. MTS assay. Data are expressed as the mean ± SEM (n= 3) and data were analysed by one-way analysis of variance (ANOVA) followed by Tukey post-hoc test. (*p<0.05)

b) D283-Med cells were treated with STAT3 inhibitor NSC 74859 (200 μM), CQ (40 μM) alone or in combination for 48h. Levels of p-STAT3 (Y705), STAT3, c-MYC, CYCLIN D2, PARP and ACTIN were analysed by WB.

c) D283-Med cells were treated as in (**b**) for different time periods and proliferation was monitored by MTS assay (upper panel) Data are expressed as the mean ± SEM (n= 6) and data were analysed by one-way analysis of variance (ANOVA) followed by Tukey post-hoc test. (*p<0.05; **p<0.01; ***p<0.001). Apoptosis was evaluated by flow-cytometry measuring Annexin V-Propidium iodure positive cells (bottom panel). Data are expressed as the mean ± SEM (n= 3) and data were analysed by one-way analysis of variance (ANOVA) followed by Tukey post-hoc test. (*p<0.05; ***p<0.001).

d) D283-Med cells were treated with WP1066 (10 μM, 48h) in the presence of CQ for 1 h. Levels of LC3 and ACTIN were analysed by WB. Densitometric analysis of LC3II levels over ACTIN is also shown (right panel). Data are expressed as the mean ± SEM (n= 3) and data were analysed by one-way analysis of variance (ANOVA) followed by Tukey post-hoc test. (*p<0.05).

e) WB analyses of p-STAT3 (Y705), c-MYC, CYCLIN D2 and ACTIN were performed in cerebellar tumours obtained from mice shown in Fig. 8**d.**

f) WB analyses of c-Myc and Ambra1 were performed in both normal cerebellum and tumours obtained from mice shown in Fig**.** 8**g**. Data are expressed as the mean ± SEM (n= 4) and were analysed by unpaired Student's t-test (*p<0.05).

Table S1: Clinical features of the patients included in this study

The tumour samples were from patients enrolled at Bambino Gesù Pediatric Hospital between 2010 and 2018

Characteristic	Measure	N°	Total Population n (%)/datum
Total Patients		47	
Age at diagnosis (years)	Median	6,7	
	Range	0.5 - 16	
Gender	Male	24	
	Female	23	
	Male/Female ratio		
Histopatological variant	Classic	30	63,82 %
	Large cell/anaplastic	10	21,27 %
	Desmoplastic/Nodular	7	14,89 %
Molecular subtype	WNT	8	17,39%
	SHH	9	19,57%
	G3	13	28,26%
	G4	16	34,78%
Status	Alive	34	
	Dead of disease	13	

SUPPLEMENTARY TABLE S1: PRIMERS qPCR

Name	species	FW	RW
AMBRA1	H	5’-AACCCTCCACTGCGAGTTGA-3’	5’-TCTACCTGTTCCGTGGTTCTCC-3’;
B2M	H	5’-CTCCGTGGCCTTAGCTGTC-3’	5’- TCTCTGCTGGATGACGTGAG-3
CD133	H	5’-CAGAGTACAACGCCAAACCA-3’	5’- AAATCACGATGAGGGTCAGC-3’;
C-MYC	H	5'-TCTCCTTGCAGCTGCTTAG-3’	5’- GTCGTAGTCGAGGTCATAG-3’;
STAT3	H	5’-ACATTCTGGGCACAAACAC-3’	5’- CTCAGTCACAATCAGGGAAG-3’
FOX03A	H	5’-AGATCTACGAGTGGATGGTG-3’	"5’-CTTGCCAGTTCCCTCATTC-3’; "
OCT-4	H	5’-TCTAGAAGTTAGGTGGGCAG-3’	"5’-CAATCTCCCCTTTCCATTCG
SOX2	H	5’-AGCTACAGCATGATGCAGGA-3’,	"5’-GGTCATGGAGTTGTACTGCA-3’;
NANOG	H	5’-TGAACCTCAGCTACAAACAG-3’	"5’-TGGTGGTAGGAAGAGTAAAG-3’;
B-III TUBULIN	H	5’-CTCAGGGGCCTTTGGACATC-3’	"5’-CAGGCAGTCGCAGTTTTCAC
ATG13	H	5’-CCCAGGACAGAAAGGACCTG-3’	5’-AACCAATCTGAACCCGTTGG-3'
GFAP	H	5’-TGGAAGCCGAGAACAACCT-3’	5'-CCTCCAGCGACTCAATCTTC-3’
SYP	H	5'-CCTCCAGCGACTCAATCTTC-3’	5'-AGCCTGTCTCCTTAAACACGAA-3’
SNAIL	H	5'-GCTGCAGGACTCTAATCCAG-3’	5'-ATCTCCGGAGGTGGGATG-3’
VIMENTIN	H	5’-TACAGGAAGCTGCTGGAAGG-3’	5'-ACCAGAGGGAGTGAATCCAG-3’
P62	H	5’-GGAGCAGATGAGGAAGATCG-3’	5'-TGGGTCCAGTCATCATCTCC-3’
MIZ-1	H	5’-CATGTCTTGGAACAGCTGAA-3’	5'-GCACTGCTTTATGAGCCTTA-3’
GADPH	H	5’-GCGAGATCCCTCCAAAATCAA-3’	5'-GTTCACACCCATGACGAACAT-3'
CYCLIN D2	H	5’-CACCGACTTTAAGTTTGCCA-3’	5'-TTGGTGATCTTAGCCAGCAG-3'
BECLIN1	H	5’-AAGAGGTTGAGAAAGGCGAG-3’	5'-TGGGTTTTGATGGAATAGGAGC-3’
NESTIN	H	5’-ATCGCTCAGGTCCTGGAA-3’	5'-AAGCTGAGGGAAGTCTTGGA-3’
SOCS3	H	5’-CCTATGAGAAAGTCACCCAG-3’	5'-TGTGCTTGTGCCATGTG-3’
ATG7	H	5’-TGAGTTGACCCAGAAGAAGCT-3’	5'-CCCAGCAGAGTCACCATTGT-3’
ATG5	H	5’-ATGTGCTTCGAGATGTGTGGT-3’	5'-AGTATGGTTCTGCTTCCCTTTCA-3’
WIPI2	H	5’-AATGCACCGATACGGAAGAT-3’	5'-GCAAACCTTTAGCTTCCTTGG-3’
LC3	H	5’-GATGTCCGACTTATTCGAGAGC-3'	5'-TTGTTTTATCCAGAACAGGAAAGC-3’
ACTINA	H	5'-GTACCACTGGCATCGTGATGGACT-3'	5'-CCGCTCATTGCCAATGGTGAT-3'
ULK1	H	5'-CAAGCTGCCCGACTTCCT-3'	5'-CTGGGAGCTGGGGGTCTT-3'
